# Simultaneous electrochemical detection of heavy metal ions using a sol–gel synthesized BiVO_4_ nanosphere modified electrode and its antimicrobial activity†

**DOI:** 10.1039/d5na00102a

**Published:** 2025-04-07

**Authors:** Keerthana Madhivanan, Raji Atchudan, Sandeep Arya, Ashok K. Sundramoorthy

**Affiliations:** a Department of Prosthodontics and Materials Science, Saveetha Dental College and Hospitals, Saveetha Institute of Medical and Technical Sciences Chennai 600077 Tamil Nadu India ashok.sundramoorthy@gmail.com; b School of Chemical Engineering, Yeungnam University Gyeongsan 38541 Republic of Korea; c Department of Physics, University of Jammu Jammu 180006 Jammu and Kashmir India

## Abstract

This study explores the development of an advanced electrochemical sensor designed for the simultaneous detection of Cd^2+^, Pb^2+^, Cu^2+^, and Hg^2+^ ions. The sensor utilizes sol–gel-synthesized bismuth vanadate (BiVO_4_) nanospheres, which are integrated onto a glassy carbon electrode (GCE), and employs square wave anodic stripping voltammetry (SWASV) for electrochemical determination of heavy metal ions. The as-prepared sensor demonstrated exceptional analytical performance and offered a wide linear detection range from 0 μM to 110 μM, along with low detection limits of 2.75 μM for Cd^2+^, 2.32 μM for Pb^2+^, 2.72 μM for Cu^2+^, and 1.20 μM for Hg^2+^ ions. These characteristics made the sensor highly suitable for precise monitoring of heavy metal contamination in both environmental and industrial samples. Beyond their sensing capabilities, the BiVO_4_ nanospheres also exhibited significant antimicrobial activity against bacterial strains such as *E. coli* and *S. aureus*, as well as fungal strains like *C. albicans* and *C. parapsilosis*. This antimicrobial effect was attributed to the enhanced surface reactivity and the generation of reactive oxygen species (ROS), which disrupt microbial cellular functions. This dual-functional approach highlighted the substantial progress in both electrochemical sensing and antimicrobial applications. This research presents a strong platform for tackling urgent challenges in environmental monitoring and microbial control.

## Abstract 2

This study explores the development of an advanced electrochemical sensor designed for the simultaneous detection of Cd^2+^, Pb^2+^, Cu^2+^, and Hg^2+^ ions.

## Graphical abstract

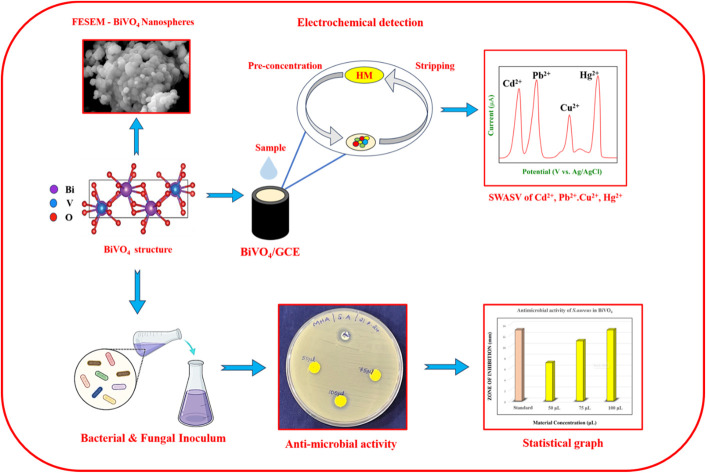

## 1. Introduction

Bismuth vanadate (BiVO_4_) is a semiconductor material that has attracted considerable attention in various scientific fields due to its exceptional photocatalytic properties, chemical stability, cost efficiency and low toxicity.^1–3^ The material's wide applicability stems from its unique electronic structure, which enables efficient light absorption and charge separation, making it ideal for applications such as photocatalysis, solar energy conversion, and environmental sensing.^1,3–7^ Among the various synthesis techniques available for BiVO_4_, the sol–gel method stands out due to its ability to produce materials with high purity, controlled morphology, and tailored surface properties.^2,8^ This method offers significant advantages in terms of fine-tuning the material's characteristics, which is crucial for optimizing performance in specific applications. The sol–gel technique is particularly effective in producing nanostructured BiVO_4_ with an enhanced surface area, making it suitable for applications requiring high sensitivity and selectivity.

The detection and quantification of heavy metals in environmental samples have become increasingly critical due to the severe health risks posed by these contaminants.^9–12^ Heavy metals such as cadmium (Cd^2+^), mercury (Hg^2+^), lead (Pb^2+^) and copper (Cu^2+^) are of particular concern because of their high toxicity, environmental persistence, and tendency to bioaccumulate in living organisms. Although copper is an essential trace element necessary for many biological processes, such as energy production, connective tissue formation, and antioxidant defence, high levels can lead to toxic effects (such as oxidative stress, mitochondrial damage, and disruption of metal homeostasis).^13^ The maximum tolerable intake of copper suggested by the WHO in drinking water is 2 mg L^−1^, whereas the limits for Hg^2+^, Pb^2+^ and Cd^2+^ are 6 μg L^−1^, 10 μg L^−1^, and 3 μg L^−1^, respectively.^14^ Chronic exposure to these metals can lead to serious health problems, including kidney damage, neurological disorders, and increased risk of cancer.^9^ As a result, there is a pressing need for reliable and sensitive methods to detect and quantify trace levels of these metals in environmental samples.

Traditional methods for detecting heavy metals, such as atomic absorption spectroscopy (AAS)^15^ and inductively coupled plasma mass spectrometry (ICP-MS),^16^ while highly accurate, are often limited by their requirement for expensive instrumentation, complex sample preparation, and the need for skilled personnel.^17^ These limitations make them less suitable for rapid, on-site environmental monitoring. In contrast, electrochemical techniques, particularly square wave anodic stripping voltammetry (SWASV), offer a promising alternative due to their simplicity, low cost, and ability to detect multiple analytes simultaneously with high sensitivity and selectivity.^17,18^

In this study, we have developed a novel electrochemical sensor based on bismuth vanadate (BiVO_4_) nanospheres synthesized through the sol–gel method, offering a highly efficient platform for the simultaneous determination of Cd^2+^, Pb^2+^, Cu^2+^, and Hg^2+^ ions. The sensor leverages SWASV to achieve sensitive and selective detection of these heavy metal ions, highlighting its potential for advanced analytical applications in environmental monitoring and toxicology. The sol–gel process allows for the fine control of BiVO_4_'s surface properties, which is critical for enhancing the material's electrochemical performance.^2^ When BiVO_4_ is used to modify the surface of an electrode, it facilitates the preconcentration of heavy metal ions from the sample solution onto the electrode surface. This preconcentration step is crucial for achieving high sensitivity in the detection process, as it enables the accumulation of metal ions at the electrode, which are then stripped off during the anodic scan in the voltammetry measurement. The result is a significant enhancement in the signal corresponding to each metal ion, allowing for the detection of Cd^2+^, Pb^2+^, Cu^2+^ and Hg^2+^ at trace levels.

The simultaneous detection of multiple heavy metals is particularly challenging due to the potential for interference between analytes and the need for selective preconcentration.^19^ However, BiVO_4_'s unique surface chemistry and electronic properties make it an excellent candidate for this task. The material's ability to form stable complexes with metal ions, combined with its high surface area, enhances its capacity for selective adsorption and preconcentration of Cd^2+^, Pb^2+^, Cu^2+^ and Hg^2+^ from complex environmental matrices.^19^ Furthermore, the use of SWASV as the detection method capitalizes on BiVO_4_'s electrochemical properties, enabling the clear resolution of voltammetric peaks corresponding to each metal ion. This approach not only ensures high sensitivity but also allows for the simultaneous quantification of Cd^2+^, Pb^2+^, Cu^2+^ and Hg^2+^ in a single analysis.

In addition to its application in environmental sensing, BiVO_4_ has garnered interest for its antimicrobial properties.^20–24^ The antimicrobial activity of BiVO_4_ is particularly relevant in the context of biomedical applications, where the development of materials that can prevent the growth and spread of pathogenic microorganisms is of great importance. BiVO_4_ has been shown to exhibit significant antimicrobial effects against a range of bacterial and fungal strains (including both Gram-positive and Gram-negative bacteria and fungi). This activity is likely due to a combination of factors, including the generation of reactive oxygen species (ROS) under light irradiation,^25^ which can damage microbial cell walls and disrupt cellular processes.^26^

The dual functionality of BiVO_4_ as both an environmental sensor and an antimicrobial agent highlights its versatility and potential for addressing some of the most pressing challenges in public health and environmental protection. In the context of environmental monitoring, the ability to simultaneously detect multiple heavy metals with high sensitivity using a BiVO_4_-modified electrode offers a practical solution for rapid, on-site analysis, which is crucial for timely decision-making in environmental management. Meanwhile, the antimicrobial properties of BiVO_4_ open up possibilities for its use in the development of antimicrobial coatings and surfaces, which could be applied in healthcare settings, food processing environments, and other areas where microbial contamination is a concern.

The sol–gel synthesis method plays a key role in unlocking these applications by enabling the production of BiVO_4_ with precisely controlled properties that are tailored to the specific needs of each application.^2^ The ability to manipulate the material's morphology, surface area, and electronic structure through the sol–gel process allows for the optimization of BiVO_4_'s performance in both electrochemical detection and antimicrobial activity. As research into the properties and applications of BiVO_4_ continues to advance, it is likely that new uses for this versatile material will emerge, further expanding its role in addressing critical challenges in environmental and public health.^2^ The sol–gel synthesized BiVO_4_ offers a multifunctional platform with significant potential for both environmental sensing and antimicrobial applications. Its use in the simultaneous electrochemical detection of Cd^2+^, Pb^2+^, Cu^2+^ and Hg^2+^ provides a practical and effective solution for monitoring heavy metal contamination in environmental samples, while its antimicrobial properties open up new avenues for preventing microbial infections. As such, BiVO_4_ represents a valuable addition to the toolkit of materials available for addressing the dual challenges of environmental contamination and microbial control, with the potential to make a significant impact in both fields.

## 2. Experimental

### 2.1. Chemicals and reagents

Mercury(ii) nitrate monohydrate was procured from ACROS Organics Pvt. Ltd. Cadmium chloride was procured from Merck, Sigma-Aldrich, India. Lead acetate, bismuth(iii) nitrate pentahydrate (Bi(NO_3_)_3_·5H_2_O) and ammonium metavanadate (NH_4_VO_3_) were purchased from SRL Chem Pvt. Ltd, India. Copper acetate was procured from Alfa Aesar Pvt. Ltd. We employed a comprehensive array of chemicals and reagents without further purification. The Cd^2+^, Pb^2+^, Cu^2+^ and Hg^2+^ solutions, along with their associated electrolytes, were prepared using double-distilled water (Milli-Q) with a resistivity of 18.2 MΩ cm. All supplementary solutions and buffers were formulated in strict accordance with established laboratory protocols.

### 2.2. Instruments

A Field Emission Scanning Electron Microscope (FESEM) (ZEISS Gemini-IIT Roorkee) was used to examine the surface morphology and structure of the BiVO_4_ nanoparticles. A three-electrode setup comprising a working electrode (glassy carbon electrode, or GCE) with a working area of 0.07 cm^2^, a counter electrode (Pt wire), and a reference electrode (Ag/AgCl soaked in 3 M KCl) was used to conduct electrochemical measurements (SWASV) using an electrochemical workstation (CHI-760E, CH Instruments, USA).

### 2.3. Sol–gel synthesis of BiVO_4_

**Fig. 1:**
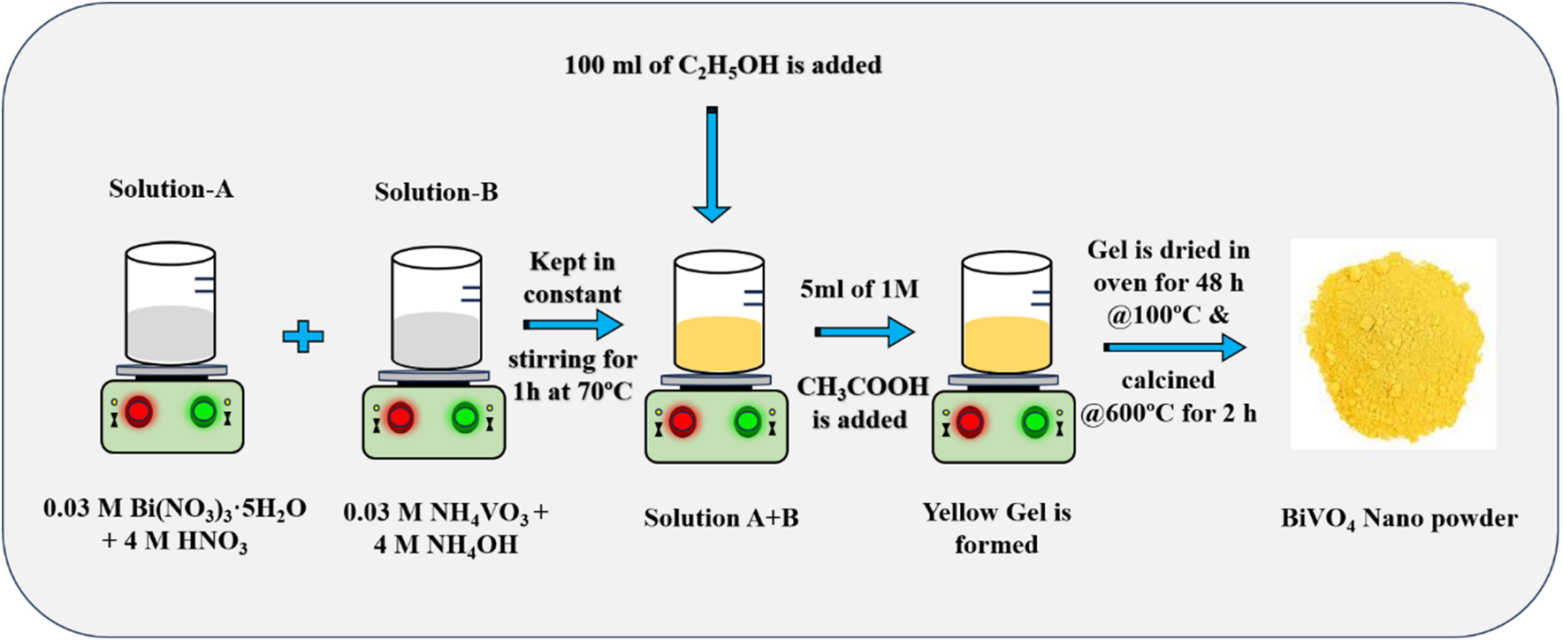
Schematic illustration of the facile sol–gel-mediated synthesis process for BiVO_4_ nanoparticles.

The preparation of BiVO_4_ powder was conducted using the sol–gel method, as schematically depicted in Fig. 1. The synthesis involved bismuth nitrate (Bi(NO_3_)_3_·5H_2_O) and ammonium vanadate (NH_4_VO_3_) as starting precursors in a 1 : 1 molar ratio. Solution A was prepared by dissolving 0.03 M Bi(NO_3_)_3_·5H_2_O in 50 mL of 4 M nitric acid (HNO_3_), while solution B consisted of 0.03 M NH_4_VO_3_ dissolved in 50 mL of 4 M ammonium hydroxide (NH_4_OH). These two solutions were combined under vigorous stirring for 30 minutes, resulting in the formation of a yellow solution. To this mixture, 100 mL of ethanol (C_2_H_5_OH) was added, and the solution was heated to 70 °C with continuous stirring for 1 hour to obtain a yellow solution. The transformation of the sol into a gel was induced by the addition of 50 mL of deionized water and 5 mL of 1 M acetic acid (CH_3_COOH), producing a yellow gel. This gel was subsequently dried in a hot air oven at 100 °C for 48 hours. The dried gel was then subjected to calcination in a muffle furnace at 600 °C for 2 h, yielding the final BiVO_4_ powder.^27^

### 2.4. Preparation of Cd^2+^, Pb^2+^, Cu^2+^ and Hg^2+^ solutions

Separate solutions of 10 mM Cd^2+^, Pb^2+^, Cu^2+^ and Hg^2+^ were made with 10 mL of DI water. To create a homogeneous solution, a five-minute bath sonication was applied to the Cd^2+^, Pb^2+^, Cu^2+^ and Hg^2+^ solutions. The Cd^2+^, Pb^2+^, Cu^2+^ and Hg^2+^ solutions were promptly covered using aluminium foil.

### 2.5. Preparation of the BiVO_4_ modified electrode

A GCE was meticulously polished using 0.05-micron alumina powder on a specialized polishing cloth to achieve a highly reflective, mirror-like finish. Following the polishing, 7 μL of the BiVO_4_ nanoparticle solution (0.5 mg mL^−1^) was precisely drop-cast onto the polished GCE surface. The modified electrode was then subjected to drying at 50 °C in a hot air oven for ten minutes. After drying, BiVO_4_/GCE was carefully retrieved and allowed to cool to room temperature. To ensure the removal of any loosely bound particles, the electrode was briefly immersed in deionized water for one minute. The prepared BiVO_4_/GCE was subsequently utilized as an electrochemical sensor for the detection of Cd^2+^, Pb^2+^, Cu^2+^ and Hg^2+^ ions. A comprehensive analysis was performed on BiVO_4_ based sensor's fabrication process, electrochemical properties, and detection capabilities to explore its potential applications in pharmaceutical quality control and environmental monitoring.

### 2.6. Characterization analyses

The BiVO_4_ nanoparticles were characterized using FESEM, EDX, EMAP, FTIR, XRD, DLS, zeta potential and UV-vis DRS spectroscopy to evaluate their morphological, structural, compositional, and optical properties. FESEM (ZEISS Gemini-IIT Roorkee) analysis revealed uniformly distributed nanoparticles with well-defined surface morphology, confirming nanoscale dimensions. EDX provided elemental composition analysis, detecting bismuth, vanadium, and oxygen in stoichiometric ratios, indicating high purity and successful formation of BiVO_4_. EMAP further validated the homogeneous distribution of these elements within the nanoparticles, confirming the uniformity of composition throughout the sample. FTIR (PerkinElmer FT-NIR) spectroscopy identified the functional groups, showing characteristic bands corresponding to Bi–O and V–O stretching vibrations, confirming the BiVO_4_ structure with no significant impurities. XRD (Bruker diffractometer) analysis provided insights into the crystalline structure, revealing sharp peaks corresponding to the monoclinic scheelite phase of BiVO_4_. Dynamic Light Scattering (DLS) analysis determined the hydrodynamic diameter of BiVO_4_ nanospheres, and zeta potential analysis indicated the surface charge of BiVO_4_ nanospheres, signifying colloidal stability and ensuring electrostatic attraction with heavy metal ions and enhanced antimicrobial efficacy by interacting with the microbial cell walls. UV-DRS (PerkinElmer lambda 365+) spectroscopy was employed to explore the optical properties, showing a distinct absorption edge typical of BiVO_4_. The band gap energy, derived from the Tauc plot, aligned with the semiconductor characteristics of BiVO_4_, underscoring its potential in photocatalytic applications. These comprehensive characterization studies affirmed the successful synthesis of high-purity BiVO_4_ nanoparticles with desirable structural and optical attributes.

### 2.7. Electrochemical analyses

The simultaneous electrochemical detection of Cd^2+^, Pb^2+^, Cu^2+^ and Hg^2+^ was achieved using a GCE modified with BiVO_4_ nanospheres. The SWASV method was conducted within a potential window of −1.0 to +0.6 V. The detection protocol was optimized at a frequency of 15 Hz, amplitude of 25 mV, increment potential of 4 mV and deposition time of 0.2 seconds, with the deposition potential set at −1.0 V. The analysis was conducted in a 0.1 M 4-(2-hydroxyethyl)-1-piperazineethanesulfonic acid (HEPES) buffer at pH 8.0, which provides a stable ionic conductive environment for metal ion detection. BiVO_4_ nanospheres exhibit a high surface area and excellent catalytic properties, enhancing the preconcentration and electrochemical response of metal ions. During the stripping step, metal ions deposited onto the electrode are oxidized, producing distinct oxidation peaks corresponding to each metal. The applied SWASV parameters ensure high sensitivity and resolution of the stripping peaks, facilitating the simultaneous quantification and making it a promising technique for environmental and industrial applications.

### 2.8. Antimicrobial activity of BiVO_4_ nanoparticles

The antimicrobial efficacy of BiVO_4_ nanoparticles was evaluated against clinical isolates of *Staphylococcus aureus*, *Escherichia coli*, *Candida albicans*, and *Candida parapsilosis*, which were collected from the Green Lab at Saveetha Dental College (SDC), Saveetha Institute of Medical and Technical Sciences (SIMATS), Saveetha University. These isolates were identified using the VITEK®2 Compact System (bioM'erieux Inc., France) at SIMATS. The bacterial strains were cultured on Mueller–Hinton Agar (MHA), which served as the medium for assessing antimicrobial activity. The antibacterial properties of the synthesized BiVO_4_ nanoparticles were investigated by measuring the zone of inhibition (ZOI) for both Gram-negative (*E. coli*) and Gram-positive (*S. aureus*) bacteria, as well as for two fungal species, *C. albicans* and *C. parapsilosis*. The ZOI is a standard metric for determining bacterial and fungal susceptibility to antimicrobial agents. In this study, the antimicrobial activity of BiVO_4_ nanoparticles was tested using MHA plates inoculated with the respective microorganisms. The bacterial and fungal cultures were grown for 24 hours, and a sterile cotton swab was used to evenly spread the inoculum across the MHA plates. Three wells, each 9 mm in diameter, were punched into MHA plates using a well cutter. These wells were then filled with different concentrations (50 μg, 75 μg, and 100 μg) of BiVO_4_ nanoparticles, which were prepared by dispersing 1 mg of BiVO_4_ in 1 mL of DMSO.^28^ Standard antibiotics were used as controls such as gentamicin for *S. aureus*, chloramphenicol for *E. coli*, and fluconazole for both *C. albicans* and *C. parapsilosis*. After allowing the samples to diffuse at room temperature for one hour, the inoculated plates were incubated at 37 °C for 24 h.^28^ Following incubation, the zones of inhibition were measured using a Hi Antibiotic Zone scale to assess the antimicrobial efficacy of the BiVO_4_ nanoparticles across different microbial species.

## 3. Results and discussion

### 3.1. FESEM, EDX and elemental mapping analyses of BiVO_4_

**Fig. 2:**
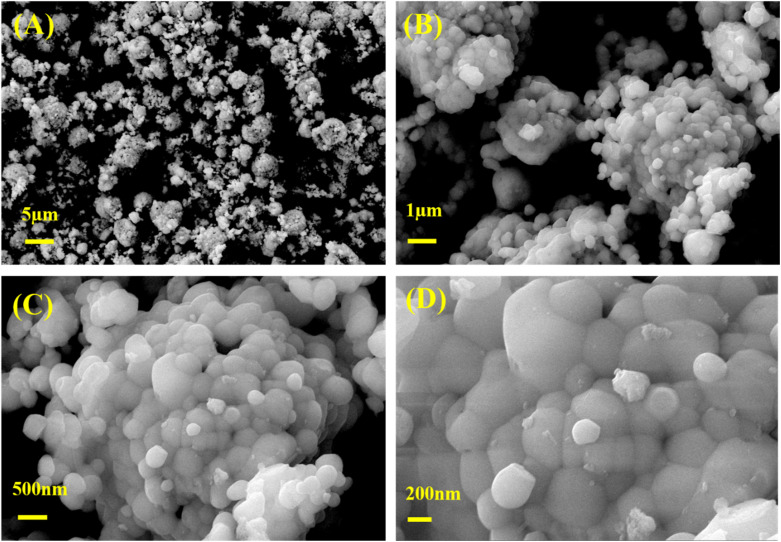
(A–D) FESEM micrographs of BiVO_4_ nanoparticles presented across different magnifications: 500 k×, 2500 k×, 5000 k×, and 10 000 k×.

Fig. 2(A–D) illustrate the morphological characteristics of sol–gel synthesized BiVO_4_ nanospheres at varying magnifications ranging from 500 k×, 2500 k×, 5000 k×, to 10 000 k×. These micrographs provided detailed insights into the morphology and surface structure of the synthesized material, demonstrating its uniformity, size distribution, and potential suitability for various applications. The particles appear predominantly spherical with a relatively uniform size distribution, indicating a successful synthesis process. They also exhibit a porous nature, suggesting an enhanced surface area, which is particularly beneficial for electrochemical, photocatalytic and photo-electrocatalytic applications. The uniformity of the nanospheres is further affirmed, with minor size deviations indicating good control over the synthesis conditions.^27^

**Fig. 3:**
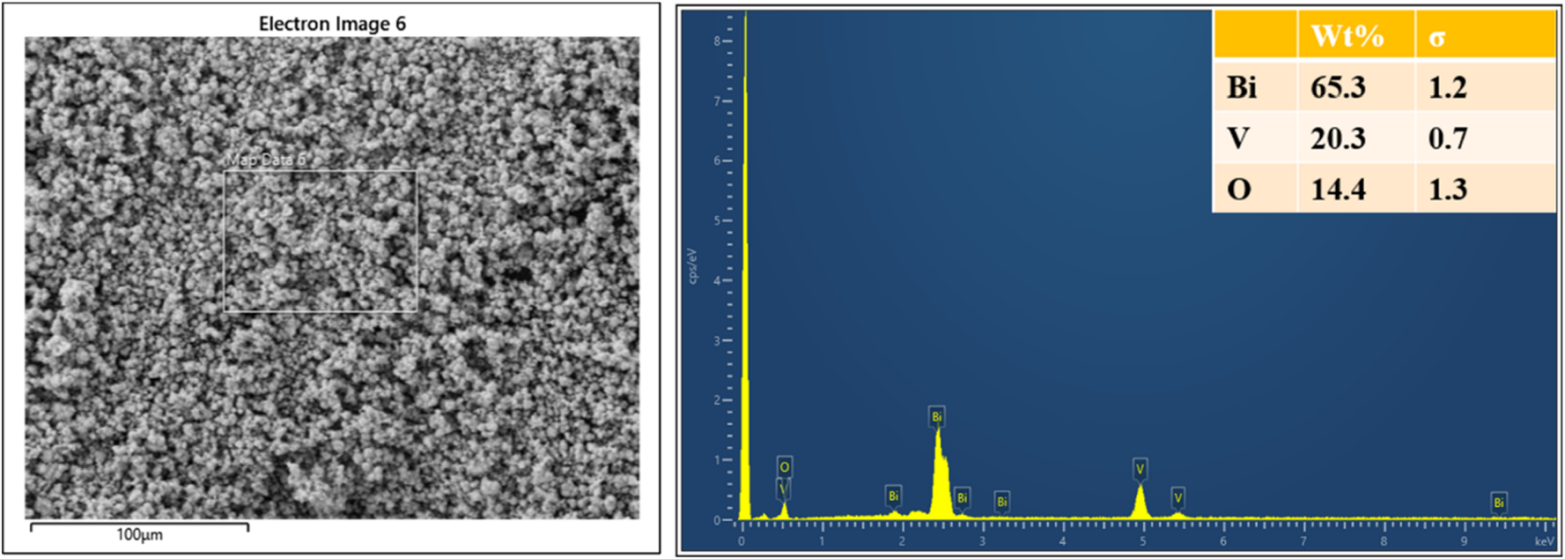
Energy-dispersive X-ray (EDX) spectrum of the synthesized BiVO_4_ nanospheres.

Overall, the FESEM images suggested that the sol–gel synthesized bismuth vanadate nanospheres exhibit a uniform spherical morphology with a porous structure. This uniformity and porosity are advantageous for various applications,^29^ where enhanced surface area and consistent particle size are critical factors. The smooth surface and consistent distribution of particle sizes reflected a well-controlled synthesis process, likely influenced by optimized parameters such as precursor concentration, pH, and calcination temperature.^2^Fig. 3 shows the EDX spectrum of BiVO_4_ nanospheres. The EDX spectrum confirmed the elemental composition of the material, showing prominent peaks corresponding to Bi (65.3 wt%), V (20.3 wt%), and O (14.4 wt%), which confirmed the formation of BiVO_4_. Bismuth, the dominant element, is indicated by strong peaks around 2.5 keV, 1.9 keV and 9.5 keV. Vanadium is also present with peaks near 0.5 and 5 keV, while oxygen is detected as part of the oxide structure.^30^ The elemental ratio closely matches the expected stoichiometry of BiVO_4_, confirming the purity and proper formation of the material. These well-defined nanospheres with a verified elemental composition highlighted the material's potential in applications such as photocatalysis and electrochemical detection.^30,31^

**Fig. 4:**
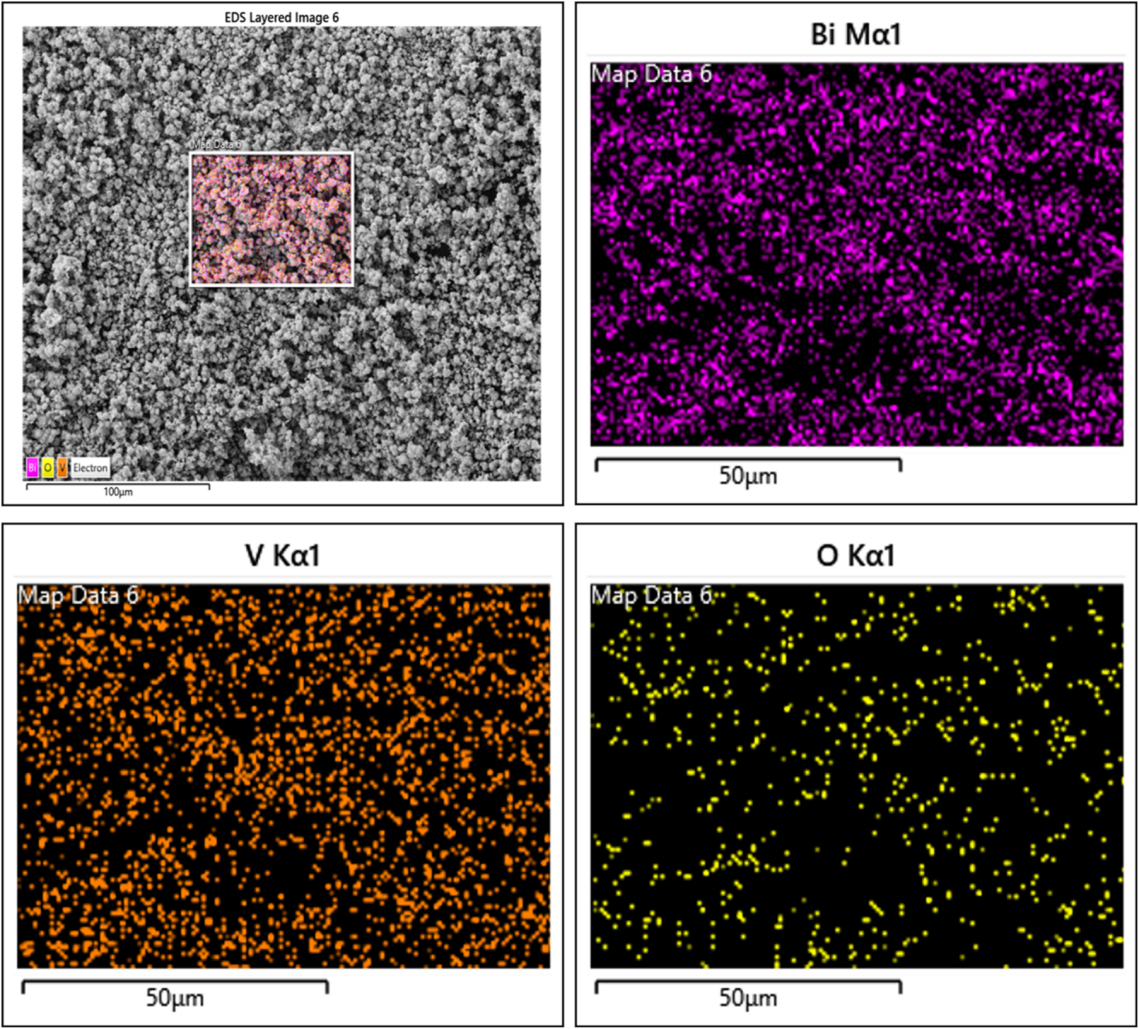
Elemental mapping representation of the synthesized BiVO_4_ nanospheres, highlighting the spatial distribution of constituent elements (bismuth (Bi), vanadium (V) and oxygen (O)).

The provided EDX elemental mapping (E-map) images illustrated the elemental distribution within sol–gel synthesized BiVO_4_ nanospheres (Fig. 4). The *E*_map_ confirmed the overall morphology of the BiVO_4_ nanospheres and their spherical structure and uniform distribution across the substrate implying the successful incorporation of (V), (Bi) and (O) into the crystal lattice, likely forming the BiVO_4_ matrix.^32^ It is a critical factor for ensuring the material's electrochemical, photocatalytic efficiency and structural integrity.^30^ The homogeneity seen across all maps highlighted the effectiveness of the sol–gel method in synthesizing uniform BiVO_4_ nanospheres.

### 3.2. DLS and ZETA potential measurements

**Fig. 5:**
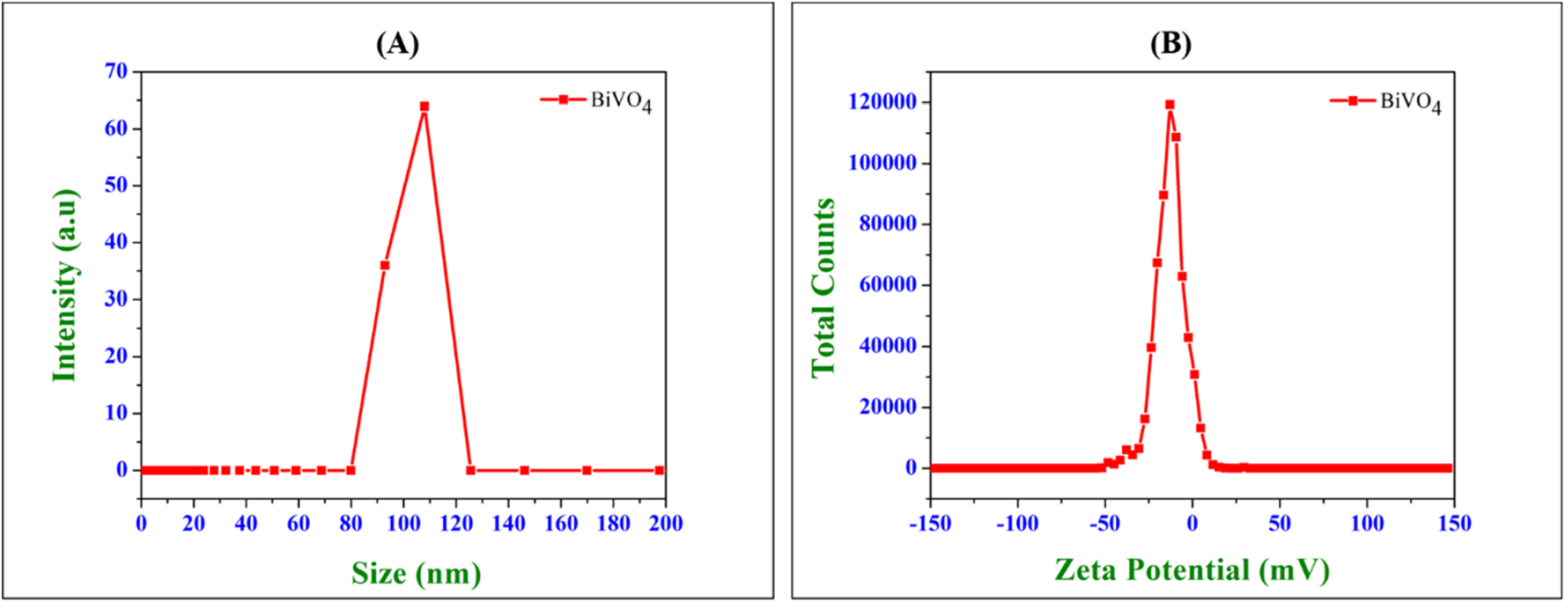
(A) DLS spectrum (particle sizes) and (B) zeta potential analysis of BiVO_4_ dispersion.

The DLS analysis (Fig. 5A) revealed a monodisperse particle distribution for the BiVO_4_ nanospheres exhibiting a hydrodynamic diameter centred at approximately 108 nm, confirming the consistent and uniform formation of the nanospheres. This nanoscale dimension plays a pivotal role in enhancing electrochemical and biological interactions.^33,34^ DLS observation exhibited a single peak, indicating that there was minimal agglomeration and a homogeneous or monodisperse distribution of BiVO_4_ nanospheres in the solution. The nanoscale size significantly amplifies the surface-to-volume ratio, promoting higher active site availability for the adsorption and electrochemical preconcentration of heavy metal ions (Cd^2+^, Pb^2+^, Cu^2+^, and Hg^2+^).^35^ This feature enhanced charge transfer kinetics, thereby improving sensor performance and the uniform size distribution ensures consistent diffusion kinetics of metal ions toward the modified electrode interface, thereby reducing mass transfer limitations and increasing analytical sensitivity.^35^

The zeta potential analysis (Fig. 5B) exhibited a sharp peak at approximately −11.7 mV, indicative of a moderately stable colloidal dispersion.^36,37^ The inherent negative surface charge of the BiVO_4_ nanospheres plays a pivotal role in both their electrochemical sensing of metal ions and antimicrobial performance. Specifically, this negative charge promotes robust electrostatic attraction with positively charged heavy metal ions (such as Cd^2+^, Pb^2+^, Cu^2+^, and Hg^2+^), thereby enhancing their preconcentration at the electrode interface and facilitating improved electron transfer kinetics. Additionally, the stable, well-dispersed colloidal state of these nanospheres minimizes aggregation, which in turn guarantees consistent and reproducible electrochemical measurements. The negatively charged BiVO_4_ nanospheres exhibit electrostatic interactions with the positively charged microbial cell walls of Gram-positive (*Staphylococcus aureus*) and Gram-negative (*Escherichia coli*) bacteria, as well as fungal strains (*Candida albicans*, *Candida parapsilosis*). This disrupts the membrane integrity, leading to cell lysis and microbial death. The visible-light-active BiVO_4_ facilitates the generation of reactive oxygen species (ROS), including superoxide anions (˙O_2_^−^) and hydroxyl radicals (˙OH), which induce oxidative stress and subsequent microbial apoptosis.^38,39^ The surface charge prevents microbial adhesion, mitigating biofilm formation—a critical factor in bacterial resistance and persistence in contaminated environments.^40^ Dynamic light scattering and zeta potential analyses yielded critical insights into the physicochemical properties of BiVO_4_ nanospheres, confirming their capacity for dual applications in heavy metal sensing and antimicrobial action. The nanometric scale combined with a moderately negative surface charge not only enhances electrochemical sensitivity for metal ion detection but also delivers formidable antimicrobial efficacy through membrane destabilization and ROS-induced cytotoxicity. These characteristics underscore the potential of BiVO_4_ as a highly promising material for environmental remediation, biosensing, and biomedical applications.

### 3.3. XRD and FTIR spectra of BiVO_4_

**Fig. 6:**
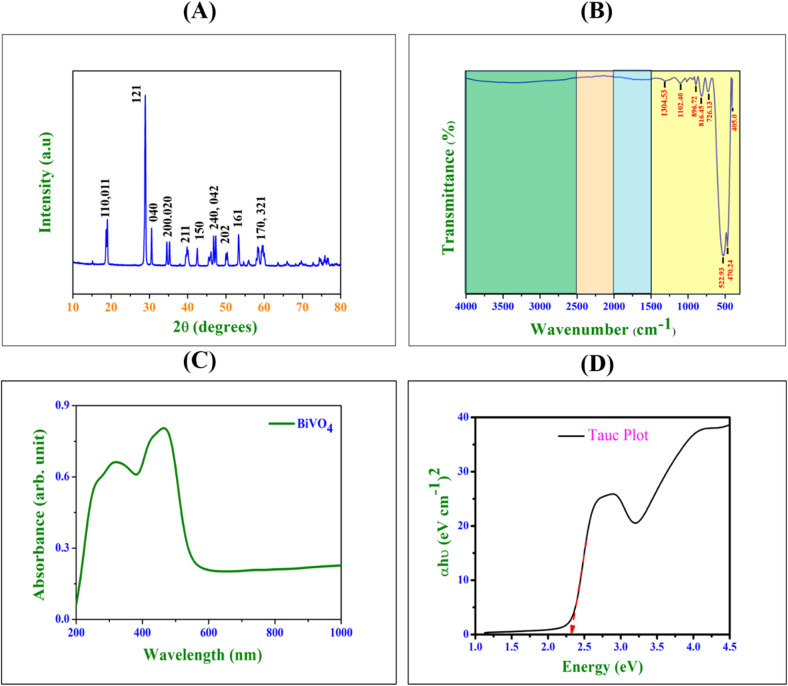
(A) XRD diffractogram of BiVO_4_ nanospheres synthesized *via* the sol–gel method, illustrating phase purity and the crystalline structure. (B) FTIR spectrum of BiVO_4_, highlighting distinct vibrational modes indicative of its chemical structure. (C) UV-vis diffuse reflectance spectrum (DRS) of the fabricated BiVO_4_ nanoparticles, detailing their optical absorbance characteristics. (D) Tauc plot of the BiVO_4_ nanoparticles, illustrating the estimation of optical band gap energy.

The XRD pattern of the sol–gel synthesized BiVO_4_ nanospheres reveals the distinct monoclinic phase of bismuth vanadate, which is well-documented under the JCPDS file number 14-0688.^41,42^ The primary diffraction peaks observed in the XRD spectrum align with known reflections of monoclinic BiVO_4_, indicating successful crystallization and phase purity as shown in Fig. 6A. The primary dominant peak at approximately 28.9° corresponds to the (121) crystallographic plane, which is the most intense reflection and serves as a hallmark of monoclinic BiVO_4_.^42^ This plane is particularly significant in determining the phase purity of the material. Other prominent peaks were also observed, which include those at 18.7° (011), 30.5° (040), 34.4° (200, 020), and 46.8° (240, 042).^42^ These diffraction planes are commonly observed in high-quality monoclinic BiVO_4_, further corroborating the successful synthesis of the desired phase.^41,42^ The appearance of minor peaks at 39.8° (211), 42.5° (150), and 53.2° (161) also confirmed the well-ordered crystalline structure.^42^ The presence of these reflections highlights the efficacy of the sol–gel method employed for the synthesis, as it ensures the formation of well-defined nanoparticles with high structural fidelity. The XRD pattern indicates that the synthesized BiVO_4_ nanospheres are in the monoclinic phase, as indicated by the prominent peaks, especially at 28.9° (corresponding to the (121) plane). The crystallinity of the material appears to be high, given the sharpness of the peaks. These findings suggest that the sol–gel synthesis method effectively produced well-ordered BiVO_4_ nanospheres with the monoclinic crystal structure.

The FTIR spectrum displayed in Fig. 6B is the characteristic of sol–gel synthesized bismuth vanadate (BiVO_4_) nanospheres, showing several distinct absorption bands that correspond to various vibrational modes of chemical bonds present in the material. This band (1304.53 cm^−1^) is attributed to the asymmetric stretching vibrations of the V–O bond in the vanadate group (VO_4_^3−^). The presence of this peak indicates the formation of the vanadate network in the material. The band at 1102.40 cm^−1^ corresponds to the stretching vibrations of Bi–O bonds in the bismuth oxide (Bi_2_O_3_) framework. The other two bands observed at 896.72 cm^−1^ and 816.45 cm^−1^ are assigned to the symmetric stretching of V–O bonds in the BiVO_4_ structure.^43^ The close proximity of these peaks further emphasizes the ordered vanadium–oxygen bonding arrangement in the material's lattice. The peak at 726.13 cm^−1^ is linked to V–O–Bi bridging vibrations, indicating the interaction between vanadate and bismuth oxide species in the nanocomposite structure.^43^ These lower wave number bands (522.93 cm^−1^ and 470.24 cm^−1^) correspond to the bending vibrations of Bi–O bonds. These bands support the presence of BiVO_4_ and its structural integrity, specifically related to the metal-oxide lattice vibrations.^43^ The band at 405.0 cm^−1^ represents the bending modes of Bi–O–V linkages in the nanospheres. It suggests a strong interaction between bismuth and vanadium oxides, typical of BiVO_4_ nanostructures.^43^ Overall, the FTIR spectrum confirmed the successful synthesis of bismuth vanadate nanospheres through the sol–gel method, showing characteristic peaks related to Bi–O and V–O bonding, which are essential components of the BiVO_4_ lattice. The distinct absorption bands affirmed the formation of a well-structured nanomaterial.

### 3.4. UV-vis DRS spectrum of BiVO_4_

The provided UV-vis diffuse reflectance spectrum (DRS) and Tauc plot of sol–gel synthesized BiVO_4_ nanospheres offer critical insights into the optical properties and band structure of the material (Fig. 6C and D). The UV-vis DRS spectrum illustrates absorbance as a function of wavelength, covering a broad range from 200 nm to 1000 nm. One prominent feature is the absorption edge, located around 500 nm, marking the transition of electrons from the valence band to the conduction band.^44^ This absorption edge is indicative of the intrinsic bandgap of BiVO_4_, signifying its capability to absorb visible light.^45^ The presence of peaks in the range of 300 to 400 nm is attributed to transitions involving surface states or defect-induced energy levels, which are commonly observed in nanostructured materials. These additional absorptions suggest the presence of sub-bandgap states or structural imperfections.^44,45^ The Tauc plot, which represents the relationship between the photon energy and the absorption coefficient (*αhν*), was used to estimate the optical bandgap of the BiVO_4_ nanospheres, as shown in Fig. 6D. The plot reveals a direct bandgap transition at approximately 2.31 eV. The sharp rise in the absorption coefficient near 2.0 eV further confirms the material's ability to absorb photons of energy corresponding to its bandgap. The broad absorption tail extending into the visible region, although weak, indicates potential contributions from defect states, suggesting that the material exhibit complex optical properties arising from its nanostructured morphology.^45^

Moreover, the UV-vis DRS spectrum and Tauc plot collectively highlight the sol–gel synthesized BiVO_4_ nanospheres as a promising material for visible light absorption, with a direct optical bandgap of around 2.31 eV. The additional spectral features suggest the presence of surface or defect-related states, which could influence the material's photocatalytic and photoelectrochemical performance. These findings underscore the potential utility of BiVO_4_ in energy conversion and environmental applications.

### 3.5. Electrochemical studies

#### 3.5.1. Effect of pH

**Fig. 7:**
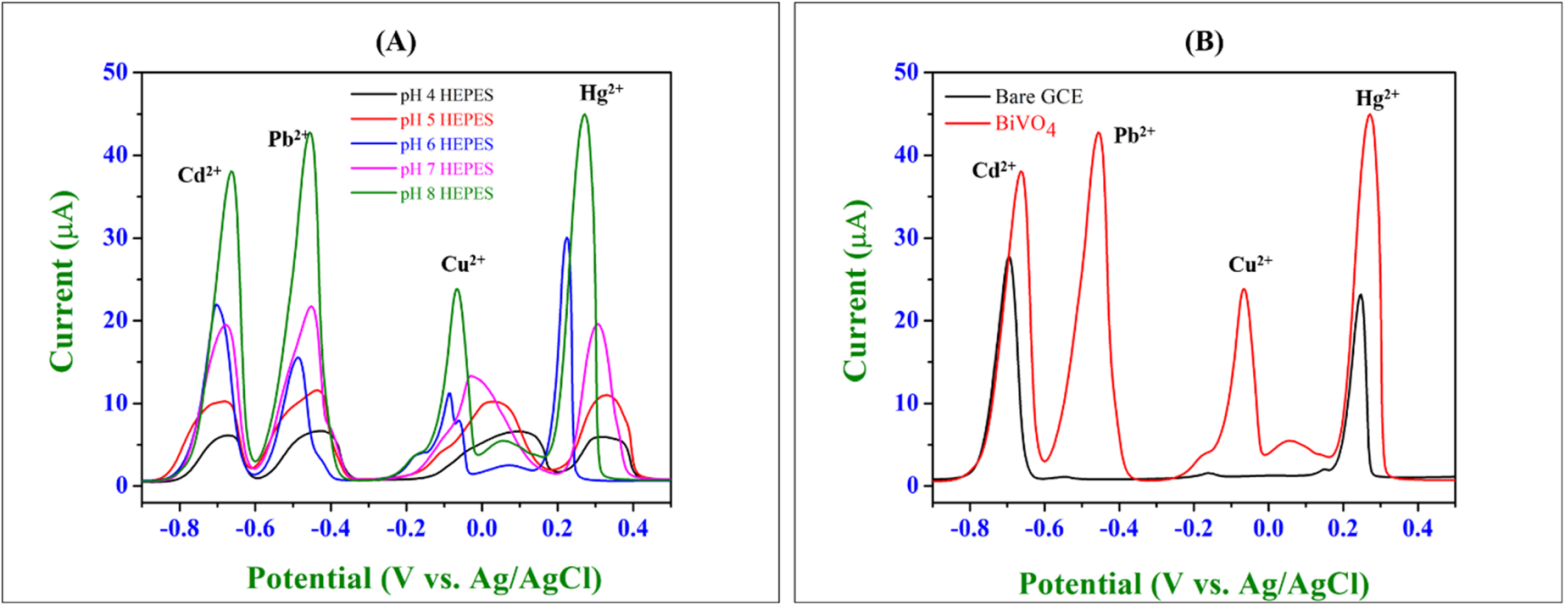
(A) SWASV voltammograms showing the effect of pH (4–8) on the BiVO_4_-modified GCE for simultaneous detection of Cd^2+^, Pb^2+^, Cu^2+^, and Hg^2+^ in 0.1 M HEPES buffer. (B) Anodic stripping voltammograms comparing the performance of the bare GCE and BiVO_4_-modified GCE for the simultaneous detection of Cd^2+^, Pb^2+^, Cu^2+^, and Hg^2+^ in 0.1 M HEPES buffer (pH 8) with an analyte concentration of 100 μM.

This study investigates the influence of pH variations in HEPES buffer on the simultaneous electrochemical detection of Cd^2+^, Pb^2+^, Cu^2+^, and Hg^2+^ using a BiVO_4_ nanosphere-modified GCE *via* SWASV (Fig. 7A). The experimental setup involves the use of 0.1 M HEPES buffer to maintain the desired pH levels (4, 5, 6, 7, and 8) while studying the electrochemical response of heavy metal ions. A fixed concentration of the analyte solution (100 μM) is added at each pH level, ensuring consistency in the metal ion concentration across the different pH conditions. The primary focus of this study was to determine the optimal pH conditions to achieve maximum sensitivity, resolution, and reproducibility for the detection of these environmentally significant heavy metals.^46^ The SWASV voltammograms indicate that both the peak current intensity and peak potential positions of heavy metal ions are highly dependent on the pH of the supporting electrolyte. The observed variations arise from the influence of pH on the adsorption–desorption dynamics, electrochemical preconcentration, and redox processes of the metal ions at the BiVO_4_-modified electrode surface.^46^ Among the tested pH levels (4–8), the optimal response was observed at pH 8 (Fig. S1†). The corresponding voltammogram exhibits well-defined, sharp, and highly resolved anodic stripping peaks with significantly higher peak currents for all metal ions. The BiVO_4_ nanospheres exhibited a negative surface charge with a zeta potential of approximately −11.7 mV. As the pH increases (4–8), the negative surface charge becomes more pronounced, enhancing the electrostatic attraction between the cationic metal ions (Cd^2+^, Pb^2+^, Cu^2+^, and Hg^2+^) and the electrode surface. At lower pH values (pH 4–5), protonation of the electrode surface reduces the electrostatic attraction, leading to diminished ion adsorption and, consequently, lower peak currents. Also, the speciation of heavy metal ions in aqueous solutions is highly pH-dependent,^46^ which may not effectively adsorb onto the BiVO_4_ surface due to competitive adsorption with protons (H^+^). The optimal pH (pH 8) leads to enhanced electron transfer kinetics due to the deprotonation of active sites on the BiVO_4_ surface, facilitating faster redox reactions at the electrode interface. The enhanced current intensity observed at pH 8 can be attributed to the increased adsorption of metal ions and improved charge transfer, leading to sharper and more distinct oxidation peaks for the analytes.^46^ This behaviour is consistent with the Nernst equation, where the potential is influenced by the activity of H^+^ ions in the electrolyte. The use of HEPES as the supporting buffer provided stable pH conditions throughout the experiments, minimizing potential drift and ensuring reproducibility of results. The buffer's p*K*_a_ value (∼7.5) makes it well-suited for maintaining mildly acidic to mildly alkaline conditions,^47^ as required for this study. pH is a critical parameter in achieving optimal electrochemical performance for heavy metal detection. Mildly alkaline conditions (pH 8) are particularly favourable for the simultaneous determination of Cd^2+^, Pb^2+^, Cu^2+^, and Hg^2+^, as they maximize the electrode's sensitivity and selectivity. The results demonstrated the significant role of pH in influencing the electrochemical behaviour of Cd^2+^, Pb^2+^, Cu^2+^, and Hg^2+^ on the BiVO_4_ nanosphere-modified GCE. The enhanced response observed at pH 8 underscores the importance of pH optimization in designing efficient electrochemical sensors. These findings pave the way for developing robust, sensitive, and reliable platforms for the real-time monitoring of toxic heavy metals in complex environmental matrices.^48^

#### 3.5.2. Anodic stripping voltammograms of Cd^2+^, Pb^2+^, Cu^2+^, and Hg^2+^

**Fig. 8:**
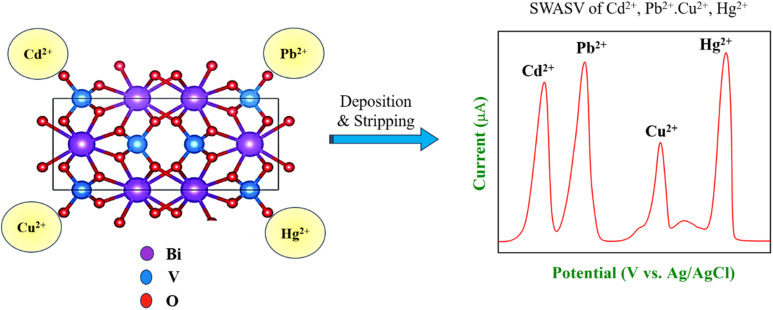
The graphical illustration of the adsorption mechanism of heavy metal ions on the surface of BiVO_4_/GCE in 0.1 M HEPES buffer determined by SWASV.^50^

The anodic stripping voltammograms illustrate the comparative electrochemical performance of a bare GCE and BiVO_4_/GCE for the simultaneous detection of Cd^2+^, Pb^2+^, Cu^2+^, and Hg^2+^ ions (Fig. 7B). BiVO_4_/GCE demonstrated significantly higher peak currents for all four metal ions (Cd^2+^, Pb^2+^, Cu^2+^, and Hg^2+^) compared to the bare GCE for analyte solution (100 μM). This enhancement highlights the superior sensitivity of the modified electrode, likely due to improved electron transfer kinetics, catalytic properties, and effective adsorption of the target analytes.^3,49^ The voltammograms exhibited well-defined and distinct peaks for each metal ion (Cd^2+^ at −0.73 V, Pb^2+^ at −0.53 V, Cu^2+^ at −0.07 V, and Hg^2+^ around 0.22 V). The clear separation of peaks minimizes signal overlap, enabling accurate simultaneous detection of the individual metal ions. The enhanced current responses observed on BiVO_4_/GCE suggest that the BiVO_4_ layer significantly improves the electrode's performance. This is likely due to its high surface area, conductivity, and electrochemical activity, which facilitate efficient preconcentration and detection of heavy metal ions.^3,49^ The use of a 0.1 M HEPES buffer at pH 8 provides a stable and optimum environment for the electrochemical detection process, supporting reliable and efficient analyte measurement. The reaction mechanism for the simultaneous detection of heavy metal ions with BiVO_4_ is shown in Fig. 8. The mechanism involves two key steps: preconcentration (reduction and deposition of ions) and stripping steps. (1) During the preconcentration step (reduction and deposition), the heavy metal ions are reduced and deposited onto the BiVO_4_-modified GCE surface in their metallic forms. The BiVO_4_ nanospheres provide a high surface area and enhanced conductivity, facilitating electron transfer and adsorption of heavy metal ions (eqn (1)–(4)).

1 Cd_(aq)_^2+^ + 2e^−^ → Cd^0^_(ads)_

2 Pb_(aq)_^2+^ + 2e^−^ → Pb^0^_(ads)_

3 Cu_(aq)_^2+^ + 2e^−^ → Cu^0^_(ads)_

4 Hg_(aq)_^2+^ + 2e^−^ → Hg^0^_(ads)_

Here, (aq) represents the aqueous phase. (ads) represents the adsorbed species on the surface of the BiVO_4_-modified electrode. The BiVO_4_ facilitates adsorption and provides catalytic sites, where the metals deposit as a thin film.

(2) Stripping step (electro-oxidation) – during the anodic scan of SWASV, the deposited metal atoms are oxidized back into their ionic forms, generating distinct oxidation currents proportional to their concentrations (eqn (5)–(8)).

5 Cd^0^_(ads)_ → Cd_(aq)_^2+^ + 2e^−^

6 Pb^0^_(ads)_ → Pb_(aq)_^2+^ + 2e^−^

7 Cu^0^_(ads)_ → Cu_(aq)_^2+^ + 2e^−^

8 Hg^0^_(ads)_ → Hg_(aq)_^2+^ + 2e^−^

The stripping currents are recorded as peaks in the voltammogram, with the peak potentials specific to each metal ion. These potentials are influenced by the electrode's catalytic properties and the stability of the metal films. The nanostructured BiVO_4_ provides abundant active sites for the adsorption and reaction of the metal ions and exhibits good conductivity, facilitating efficient charge transfer between the electrode and solution. BiVO_4_ stabilizes the reduced forms of the metal ions during the preconcentration step, ensuring better sensitivity. Hence, the BiVO_4_-modified GCE exhibits markedly improved sensitivity compared to the bare GCE for the simultaneous detection of Cd^2+^, Pb^2+^, Cu^2+^, and Hg^2+^ ions. These findings underline its potential as an effective electrode material for environmental monitoring and trace-level detection of heavy metals.

#### 3.5.3. Linear detection of Cd^2+^, Pb^2+^, Cu^2+^ and Hg^2+^

**Fig. 9:**
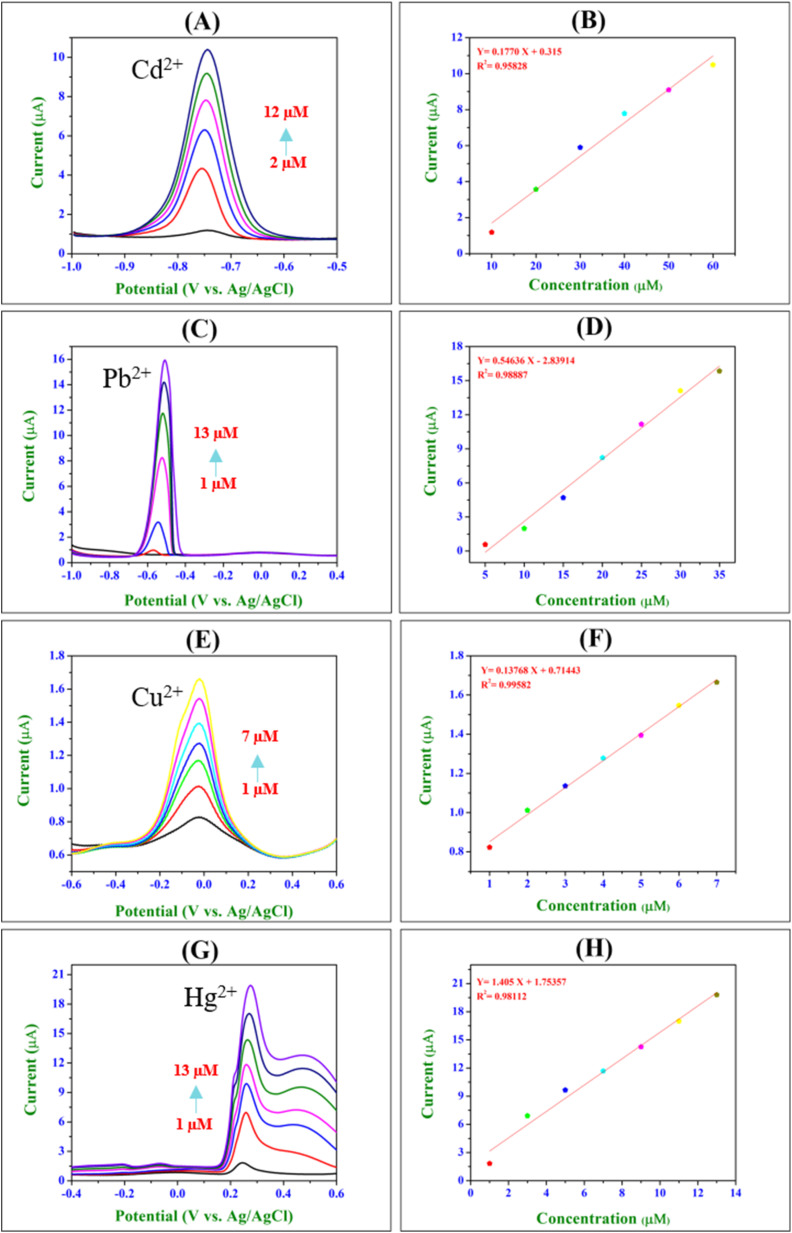
The SWASV voltammograms for detection of individual Cd^2+^, Pb^2+^, Cu^2+^, and Hg^2+^ at various concentrations using BiVO_4_/GCE. (A) 2 μM to 12 μM Cd^2+^, (C) 1 μM to 13 μM Pb^2+^, (E) 1 μM to 7 μM Cu^2+^, (G) 1 μM to 13 μM Hg^2+^. The linearity plots showing the anodic peak currents *versus* concentrations of all individual heavy metal ions [(B) Cd^2+^, (D) Pb^2+^, (F) Cu^2+^, and (H) Hg^2+^].

The electrochemical detection of individual heavy metal ions (Cd^2+^, Pb^2+^, Cu^2+^, and Hg^2+^) using SWASV on the BiVO_4_-modified GCE is depicted in Fig. 9(A, C, E and G). The voltammograms exhibited distinct and well-defined anodic peaks at the respective reduction potentials of Cd^2+^ (−0.74 V), Pb^2+^ (−0.50 V), Cu^2+^ (−0.018 V), and Hg^2+^ (0.27 V) *versus* Ag/AgCl. A progressive increase in peak current is observed with the rise in metal ion concentrations, indicating a robust correlation between current intensity and analyte concentration.^51^ This linear enhancement in peak current reflects the efficient adsorption and accumulation of metal ions on the electrode surface, driven by the negative surface charge of BiVO_4_ nanospheres. The corresponding calibration plots (Fig. 9B, D, F and H) exhibited excellent linearity between peak current and concentration across the tested range, with a correlation coefficient (*R*^2^) of 0.98, underscoring the high sensitivity, precision, and reproducibility of the developed sensor. The linear equations obtained for each metal ion are Cd^2+^: *Y* = 0.1770*X* + 0.315, *R*^2^ = 0.95828; Pb^2+^: *Y* = 0.5436*X* − 2.83914, *R*^2^ = 0.98887; Cu^2+^: *Y* = 0.13768*X* + 0.71443, *R*^2^ = 0.99582; and Hg^2+^: *Y* = 1.405*X* + 1.75357, *R*^2^ = 0.98112.

The individual detection results corroborated the findings from the simultaneous detection of these ions, wherein similar electrochemical behaviour and linear responses were observed, which confirmed the sensor's high selectivity and sensitivity.^51^ These results validated the suitability of the BiVO_4_-modified GCE for reliable and reproducible detection of heavy metal ions in both individual and mixed analyte environments.

**Fig. 10:**
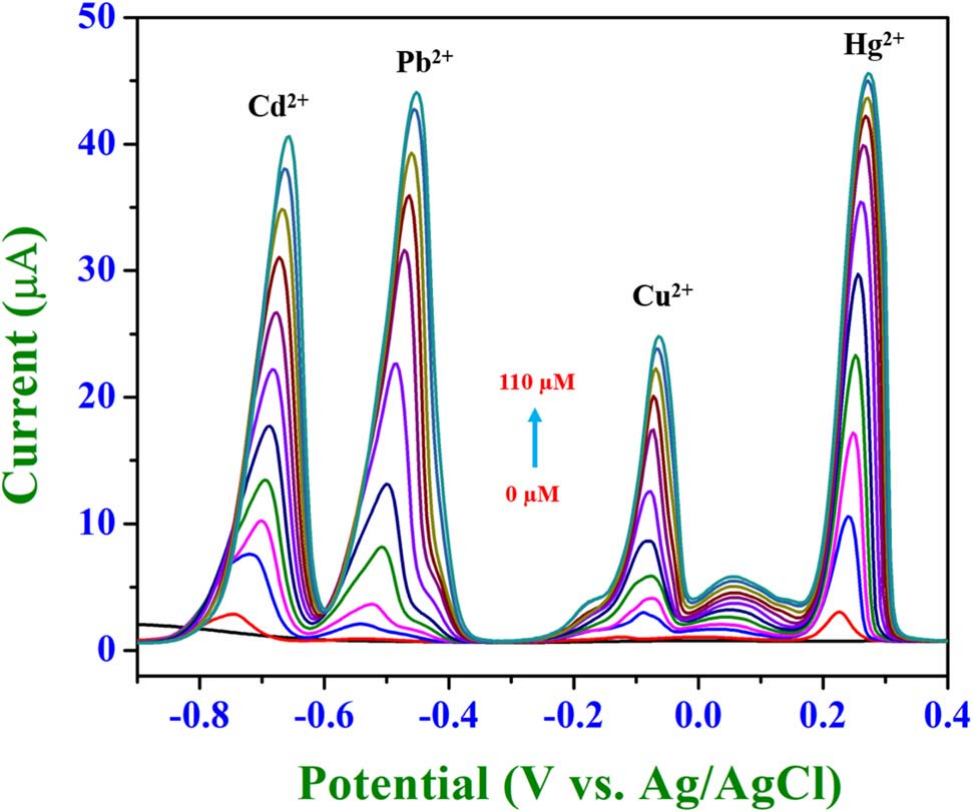
SWASV voltammograms for the simultaneous detection of Cd^2+^, Pb^2+^, Cu^2+^ and Hg^2+^ using BiVO_4_/GCE. Anodic peaks corresponding to each metal are observed in the concentration range of 0–110 μM.

The presented SWASV results illustrated the simultaneous detection of Cd^2+^, Pb^2+^, Cu^2+^ and Hg^2+^ within the concentration range of 0 μM to 110 μM using BiVO_4_/GCE (Fig. 10).

The voltammogram displays four well-resolved anodic peaks at distinct potentials, each corresponding to the oxidation of metal ions (Cd^2+^ at −0.73 V, Pb^2+^ at −0.53 V, Cu^2+^ at −0.07 V, and Hg^2+^ at 0.22 V). This demonstrated the high selectivity of the BiVO_4_-modified GCE, effectively differentiating between the target metal ions in a complex matrix. The peak current intensities exhibited a systematic and proportional increase with rising analyte concentrations, validating the linearity of the electrochemical response. This behaviour confirms the electrode's capability for accurate quantification over the specified range.^48^ The BiVO_4_ modification significantly amplified the stripping currents for all four metals. This enhancement can be attributed to the material's superior physicochemical properties, including its high specific surface area, excellent catalytic activity, and efficient electron transfer kinetics.^3,49^ These attributes facilitate robust metal ion adsorption and pre-concentration on the electrode surface. The well-defined peak shapes and their consistent growth across the concentration range reflect the electrode's reproducibility and sensitivity, ensuring reliable detection even at low metal ion concentrations. These data strongly support the BiVO_4_-modified GCE as a promising platform for advanced electrochemical sensing applications, particularly for the simultaneous detection of multiple toxic heavy metals.^3,49^

The linearity plots presented represent the calibration curves for the simultaneous detection of Cd^2+^, Pb^2+^, Cu^2+^ and Hg^2+^ using BiVO_4_/GCE (Fig. S2†). Each plot demonstrated the relationship between the anodic stripping peak current and the concentration of the respective metal ions in the range of 0 μM to 110 μM. Herein, all four calibration curves exhibited a strong linear relationship between current and concentration, with regression coefficients (*R*^2^) close to unity and these values indicated excellent linearity, confirming the high accuracy of the modified electrode for quantitative detection across the studied concentration range.^46^ The calculated LOD values demonstrated the sensor's capability to detect low concentrations of heavy metals Cd^2+^: 2.75 μM, Pb^2+^: 2.32 μM, Cu^2+^: 2.72 μM and Hg^2+^: 1.20 μM, respectively. Meanwhile, the reproducibility and consistency of the calibration curves with increasing concentration suggested uniform electrode activity across all analytes, further supporting the reliability of the sensor for multi-metal analysis.^51^ Table S1† provides a detailed comparison of recent electrochemical sensors utilizing nanoparticles for heavy metal analysis focussing on their design, detection methods, sensitivity, and selectivity. Key performance metrics such as the linear range and limits of detection are highlighted and compared with our work.^52–58^

The interference of heavy metal ions (Cd^2+^, Pb^2+^, Cu^2+^, and Hg^2+^) was rigorously investigated using SWASV with a BiVO_4_-modified GCE, as depicted in Fig. S3(A and B).† In Fig. S3(A),† the concentrations of Cd^2+^ and Pb^2+^ were systematically increased from 50 μM to 70 μM, while the concentrations of Cu^2+^ and Hg^2+^ were held constant at 50 μM. The resulting voltammograms exhibited a progressive increase in the anodic peak currents of Cd^2+^ (−0.74 V) and Pb^2+^ (−0.50 V), with no significant alteration in the peak intensities or positions of Cu^2+^ (−0.06 V) and Hg^2+^ (0.27 V). This indicates that the increased concentrations of Cd^2+^ and Pb^2+^ exert negligible interference on the electrochemical response of Cu^2+^ and Hg^2+^, thereby preserving the sensor's selectivity under these conditions. Conversely, Fig. S3(B)† illustrates the scenario where Cu^2+^ and Hg^2+^ concentrations were increased from 50 μM to 70 μM, while maintaining Cd^2+^ and Pb^2+^ concentrations held constant at 50 μM. In this case, a pronounced enhancement in the anodic peak currents of Cu^2+^ and Hg^2+^ is observed, with minimal impact on the anodic peak profiles of Cd^2+^ and Pb^2+^, affirming the sensor's resilience against cross-interference.^51^ The consistent peak separation and stability of the sensor response in both scenarios highlighted the exceptional anti-interference capability of the BiVO_4_-modified GCE, which effectively discriminates and quantifies individual ions, even in the presence of elevated concentrations of competing species.^51^ These findings underscore the robustness and high selectivity of the BiVO_4_-modified sensor, establishing its suitability for the simultaneous detection of multiple heavy metal ions in complex environmental matrices without compromising analytical accuracy.^48^

#### 3.5.4. Repeatability, reproducibility and stability analysis

Fig. S4(A)† illustrates the SWASV responses for the detection of a 75 μM analyte using a GCE modified with 7 μL of BiVO_4_ dispersion. The electrode was immersed in the analyte solution, followed by rinsing with deionized water to remove any residual analyte, and subsequently used for sensing in the 75 μM analyte solution. This process was repeated three times (*N* = 1, *N* = 2, and *N* = 3) to evaluate the repeatability of the modified electrode. The overlapping voltammograms exhibited consistent peak positions and peak intensities corresponding to Cd^2+^, Pb^2+^, Cu^2+^, and Hg^2+^, confirming the high repeatability of the sensor. Minimal variation in current responses during successive measurements suggests the high stability and robustness of the BiVO_4_-modified GCE during repetitive sensing. Fig. S4(B)† presents a bar graph quantifying the current responses for each analyte during the three repetitions. The negligible deviation between the three measurements reaffirmed the reproducibility of the sensor response under identical experimental conditions.

Fig. S4(C)† depicts the SWASV profiles of five independently modified GCEs, each prepared by depositing 7 μL of BiVO_4_ dispersion and subsequently used to detect 75 μM of analyte (Cd^2+^, Pb^2+^, Cu^2+^ and Hg^2+^) under the same conditions. The consistency of the voltammetric signals across different electrodes highlighted the reproducibility of the sensor fabrication process. The overlapping nature of the peak currents for all the analytes confirmed that the modification process yields uniform and reproducible electrode surfaces. Fig. S4(D)† shows a comparative bar graph of the current responses for Cd^2+^, Pb^2+^, Cu^2+^, and Hg^2+^ across the five independently prepared electrodes. The relative standard deviation (RSD) values, derived from the reproducibility data, provide a quantitative measure of the sensor's reproducibility. As shown in Table S2,† the RSD values provide insight into the reproducibility of the current response across independently prepared electrodes. The lower RSD values for Pb^2+^ (2.61%) and Cd^2+^ (8.45%) indicate high reproducibility, while the slightly higher RSD values for Cu^2+^ (12.37%) and Hg^2+^ (8.89%) suggest minor variability, which may be attributed to differences in surface modification or slight variations in analyte adsorption during multiple electrode preparations.

The repeatability study demonstrated the sensor's robustness and consistent performance over multiple measurement cycles using the same modified electrode, while the reproducibility study confirmed the uniformity and reliability of the sensor fabrication process across multiple modified electrodes. The calculated RSD values further validated the high reproducibility of the BiVO_4_-modified GCE, ensuring reliable and consistent detection of heavy metal ions.

The stability of BiVO_4_/GCE was assessed by performing 50 consecutive SWASV cycles in the absence of any analyte. The current response at a specific potential was monitored and recorded after every 5 cycles to evaluate the electrochemical stability of the modified electrode. As depicted in the stability plot (Fig. S4(E)†), the current response exhibited negligible fluctuation over the course of 50 cycles, demonstrating consistent electrochemical performance with minimal signal degradation. The slight variations observed after successive cycles can be attributed to minor surface restructuring or electrochemical conditioning of the BiVO_4_ layer. However, the overall retention of the current response throughout the study confirmed that the BiVO_4_ modification remains stable under prolonged electrochemical cycling, indicating robust adhesion of the nanomaterial to the electrode surface and exceptional electrochemical durability. The high stability of BiVO_4_/GCE suggests its potential applicability in long-term electrochemical sensing applications, where sustained sensor performance is critical.

### 3.6. Antimicrobial efficacy of BiVO_4_

**Fig. 11:**
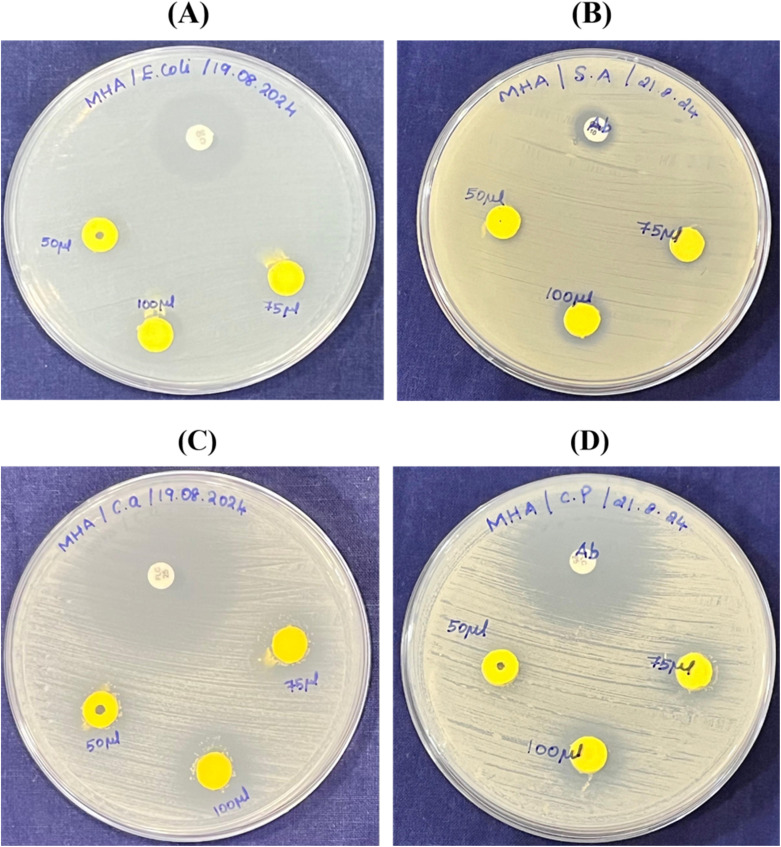
(A–D) Antimicrobial activity of sol–gel-synthesized BiVO_4_ nanospheres evaluated against selected microorganisms. (A – *E. coli*; B – *S. aureus*; C – *C. albicans*; and D – *C. parapsilosis*).

The antimicrobial efficacy of BiVO_4_ nanospheres against the tested microorganisms was assessed by measuring the zones of inhibition (ZOI) (Fig. 11A–D). The inhibition zones of BiVO_4_ nanospheres were then compared to those of standard antibiotics. The BiVO_4_ nanospheres demonstrated significant antibacterial activity against the microorganisms. Table 1 presents the ZOI measurements for both the BiVO_4_ nanospheres and the standard antibiotics, highlighting their relative antimicrobial performance.

**Table 1:** The comparison of the ZOI of standard antibiotics and BiVO_4_ nanospheres

Microorganisms	Zone of inhibition			
	Standard antibiotics	BiVO_4_ nanospheres (μg)		
		50 μg	75 μg	100 μg
*S. aureus*	Gentamicin = 13 mm	7 mm	11 mm	13 mm
*E. coli*	Chloramphenicol = 20 mm	11 mm	13 mm	15 mm
*C. parapsilosis*	Fluconazole = 30 mm	11 mm	13 mm	15 mm
*C. albicans*	Fluconazole = 29 mm	7 mm	12 mm	15 mm

For *E. coli*, inhibition zones of 11 mm (50 μg), 13 mm (75 μg) and 15 mm (100 μg) were observed, increasing with the concentration of BiVO_4_ nanospheres. *E. coli*, a Gram-negative bacterium, possesses an outer membrane enriched with lipopolysaccharides (LPSs), which confers a negative surface charge.^59^ The interaction of BiVO_4_ nanospheres, which exhibit a negative zeta potential of approximately −11.70 mV, suggests that electrostatic repulsion may limit direct attachment to the bacterial membrane. However, the antimicrobial activity arises predominantly through ROS generation. Upon exposure to BiVO_4_, photocatalytic activation under light irradiation generates hydroxyl radicals (˙OH), superoxide anions (O_2_^−^˙), and singlet oxygen (^1^O_2_),^20,60^ which initiate oxidative stress and lipid peroxidation, leading to disruption of membrane integrity, protein denaturation, and eventual cell lysis. For *S. aureus*, the ZOI ranged from 7 mm (50 μg), 11 mm (75 μg) to 13 mm (100 μg). *S. aureus* possesses a thick peptidoglycan layer in its cell wall, which imparts a positive surface charge,^61,62^ thereby facilitating a stronger electrostatic attraction toward the negatively charged BiVO_4_ nanospheres.^63^ This interaction enhances the binding affinity of the nanospheres to the bacterial surface, promoting localized ROS accumulation and oxidative stress.^63^ The generated ROS initiate lipid peroxidation, resulting in membrane destabilization, leakage of intracellular content, and subsequent bacterial death.^63^

For *C. parapsilosis*, the ZOI ranged from 11 mm (50 μg), 13 mm (75 μg) to 15 mm (100 μg). *C. parapsilosis*, a non-albicans *Candida* species, is known for its biofilm formation capabilities and resistance to conventional antifungal agents.^64^ BiVO_4_ nanospheres disrupt fungal growth through ROS generation, resulting in oxidative damage to fungal membrane lipids and intracellular components. Additionally, ROS accumulation induces apoptosis-like cell death by disrupting mitochondrial function and inducing DNA fragmentation.^63^ Similarly for *C. albicans*, the inhibition zones of 7 mm, 12 mm, and 15 mm were observed for increasing concentrations of BiVO_4_ nanospheres. *C. albicans*, a major cause of invasive candidiasis, exhibits a negatively charged cell wall composed of β-glucans and chitin.^65^ Similar to *C. parapsilosis*, the antimicrobial activity of BiVO_4_ against *C. albicans* is primarily driven by ROS-mediated oxidative stress, leading to membrane destabilization and increased permeability. The ROS-induced oxidative damage causes depolarization of mitochondrial membranes, resulting in apoptosis-like programmed cell death.^66^

The statistical graph Fig. S5(A–D)† clearly indicated the BiVO_4_ nanospheres that exhibit excellent antimicrobial activity against the microorganisms. BiVO_4_ nanoparticles are known for their photocatalytic properties, leading to oxidative stress in microbial cells and disruption of cellular structures; also, the small size and high surface-to-volume ratio of BiVO_4_ nanospheres enhance their interaction with bacterial and fungal membranes, causing structural damage. Hence, the dose-dependent response of BiVO_4_ suggests scalability and adaptability for therapeutic applications, especially as alternative agents against drug-resistant microorganisms.^28,66^ The observed correlation between the electrochemical sensing efficiency and antimicrobial performance of BiVO_4_ nanospheres can be ascribed to their exceptional physicochemical attributes. The surface area and negative surface charge of BiVO_4_ facilitated enhanced adsorption of analytes and promoted rapid electron transfer, resulting in superior electrochemical sensitivity and selectivity. Concurrently, these characteristics contribute to the generation of reactive oxygen species (ROS), such as hydroxyl radicals (˙OH) and superoxide ions (O_2_˙^−^), which induce oxidative stress and disrupt microbial cell membranes, thereby imparting potent antimicrobial activity.^20,60,63^ Additionally, the electrostatic interactions between the negatively charged BiVO_4_ surface and the positively charged microbial cell membranes facilitate stronger microbial adhesion, accelerating cell membrane disruption and microbial inactivation. This dual functionality underscores the multifunctional efficacy of the BiVO_4_-modified sensor, establishing it as a promising candidate for simultaneous electrochemical detection and antimicrobial applications in environmental and biomedical domains.

Future work will explore the development of composites with advanced nanomaterials such as graphene oxide or functionalized carbon nanotubes to achieve ultra-low detection limits, meeting stringent environmental and industrial standards.^48,67^ The sensor platform holds significant potential for integration into portable, field-deployable devices for real-time monitoring of heavy metal contamination in diverse environments such as water bodies, industrial effluents, and food products.^48,68^ Expanding the antimicrobial studies to include a broader range of pathogens, including multi-drug-resistant bacterial and fungal strains, would enhance the applicability of the findings in clinical, pharmaceutical, and environmental microbiology.^69^ Future efforts should focus on improving the long-term stability and reusability of the bismuth vanadate nanosphere-modified electrodes. Employing surface coatings or composite materials could extend the sensor's lifespan and efficiency under varied environmental conditions. Coupling the developed sensor with adsorbent materials for simultaneous detection and removal of heavy metals can offer a dual-purpose solution for water purification and environmental remediation. Incorporating computational techniques such as machine learning and chemometric modelling can refine signal interpretation and improve the differentiation of overlapping signals in complex, mixed-metal solutions.^70^ Scaling up the synthesis of sol–gel-synthesized bismuth vanadate nanospheres and automating electrode fabrication could facilitate mass production, paving the way for commercialization and widespread adoption in environmental monitoring systems. Beyond heavy metal detection, the electrochemical platform could be adapted for detecting other analytes, such as biomolecules or pharmaceutical residues, by functionalizing the electrode surface with selective recognition elements.^66^

## 4. Conclusion

This study presented a breakthrough in the development of a highly sensitive and dependable electrochemical sensor for the simultaneous detection of Cd^2+^, Pb^2+^, Cu^2+^, and Hg^2+^ ions, leveraging sol–gel-synthesized bismuth vanadate nanospheres. The incorporation of square wave anodic stripping voltammetry ensures accurate quantification within a practical concentration range, underscoring the sensor's applicability to environmental and industrial monitoring. Additionally, the investigation of antimicrobial activity against prominent bacterial and fungal species demonstrated the materials' multifunctionality and adaptability. By addressing current challenges and capitalizing on proposed future directions, this research provides a pathway for substantial advancements in environmental remediation, clinical diagnostics, and sustainable material innovation. The findings establish a comprehensive platform with the capacity to tackle global challenges in pollution control, microbial management, and beyond.

## Data availability

We provided all the obtained data in the manuscript itself.

## Conflicts of interest

There are no conflicts to declare.

## Supplementary Material

NA-OLF-D5NA00102A-s001
