# Supplementary material for: Simultaneous electrochemical detection of heavy metal ions using a sol–gel synthesized BiVO4 nanosphere modified electrode and its antimicrobial activity
Source: Nanoscale Adv. 2025 Apr 7;7(11):3432–48. doi: 10.1039/d5na00102a (PMC12013505; doi:10.1039/d5na00102a)
Supplement: NA-007-D5NA00102A-s001 [file NA-007-D5NA00102A-s001.pdf]

Supplementary Information (SI) for Nanoscale Advances.
This journal is © The Royal Society of Chemistry 2025

Supplementary Information

Simultaneous Electrochemical Detection of Heavy Metal Ions Using a Sol-Gel Synthesized $BiVO_4$ Nanospheres Modified Electrode and Their Antimicrobial Activity

Keerthana Madhivanan[1,a], Raji Atchudan[2,a], Sandeep Arya[3] and Ashok K. Sundramoorthy[1,*]

[1]Department of Prosthodontics and Materials Science, Saveetha Dental College and Hospitals, Saveetha Institute of Medical and Technical Sciences, Chennai, 600077, Tamil Nadu, India

[2]School of Chemical Engineering, Yeungnam University, Gyeongsan 38541, Republic of Korea

[3]Department of Physics, University of Jammu, Jammu 180006, Jammu and Kashmir, India

*Corresponding author: Email: ashok.sundramoorthy@gmail.com

[a] These authors are equally contributed.

List of Supporting Figures

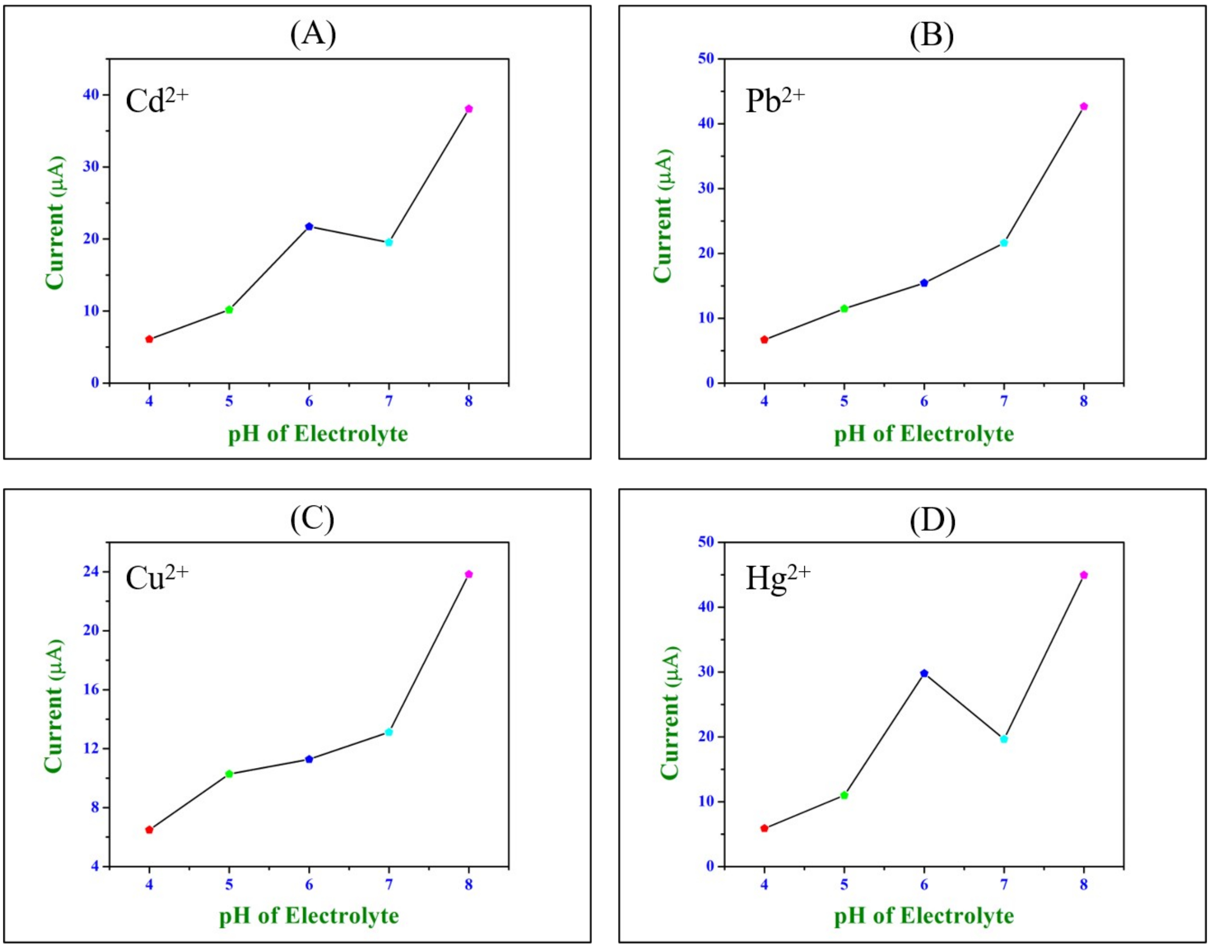

Figure S1. The graphical representation of anodic peak current (I_{pa}) vs. pH (pH 4 – 8) of the buffer for electrocatalytic oxidation of heavy metal ions (100 μM) on $BiVO_4$/GCE.

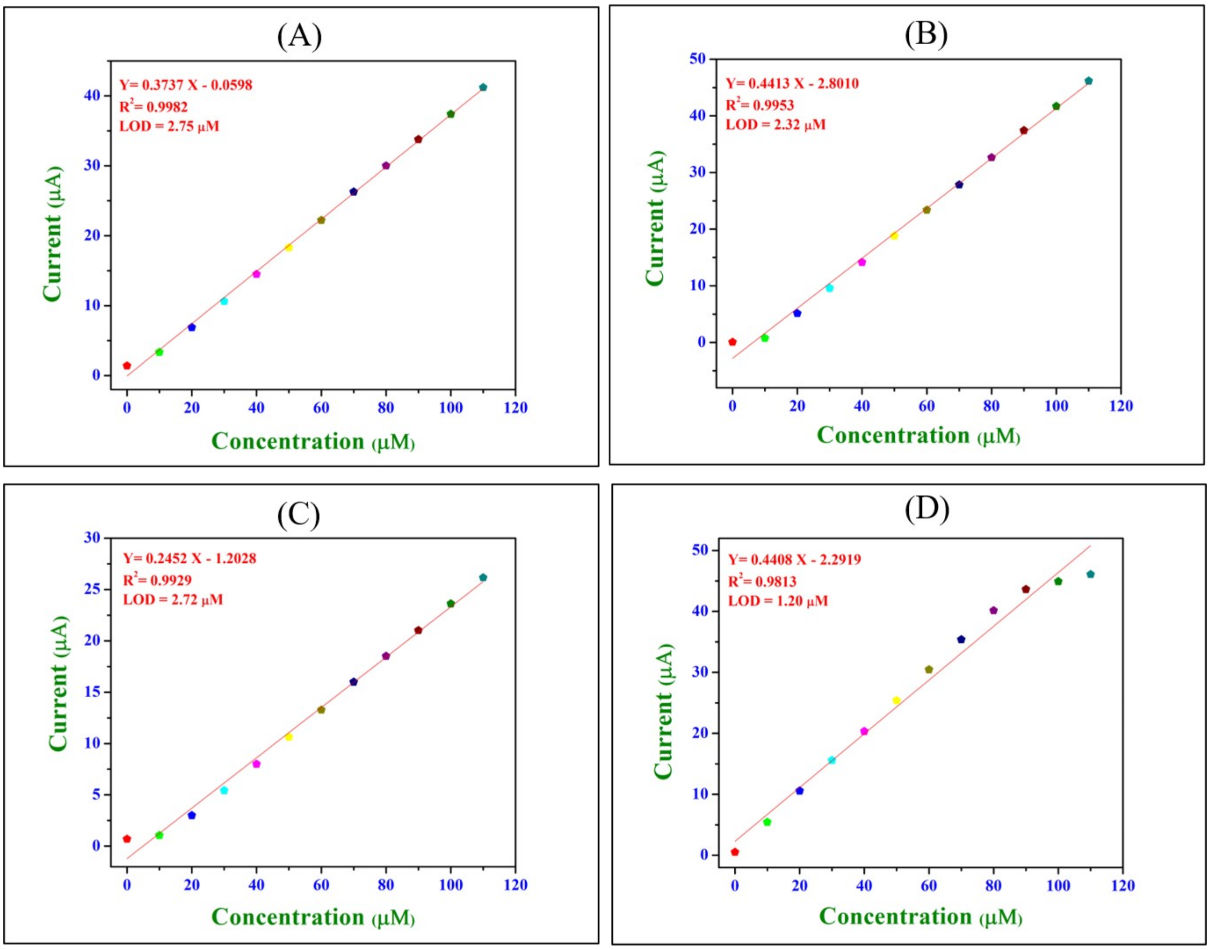

Figure S2. Linearity plots showing the anodic peak currents versus concentrations (0 – 110 µM) for Cd^{2+} (A), Pb^{2+} (B), Cu^{2+} (C), and Hg^{2+} (D) using a $BiVO_4$-modified GCE.

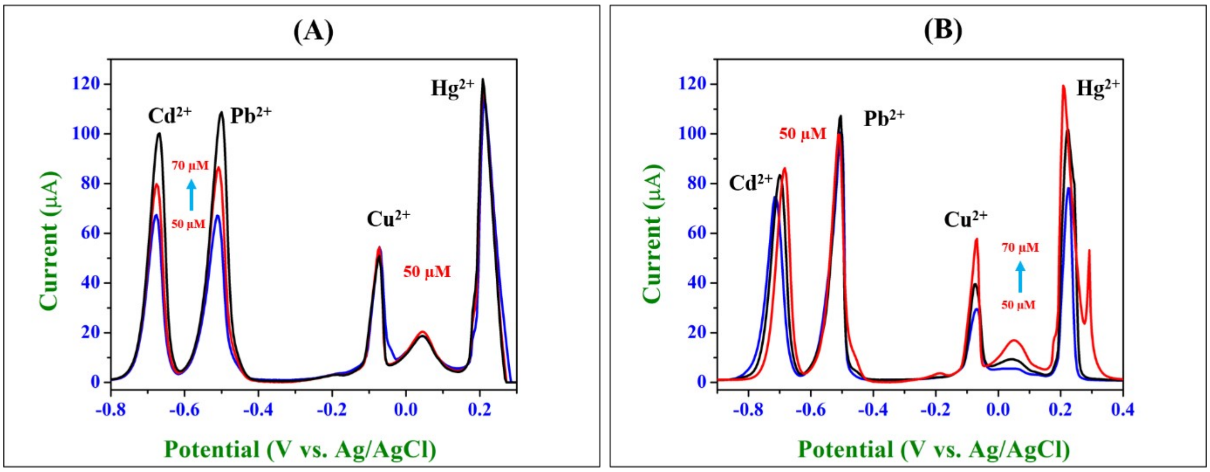

Figure S3. SWASV voltammograms illustrating the mutual interference analysis of heavy metal ions, demonstrating the sensor's capability to maintain distinct and well-defined anodic peaks with minimal cross-interference, even under varying analyte concentrations.

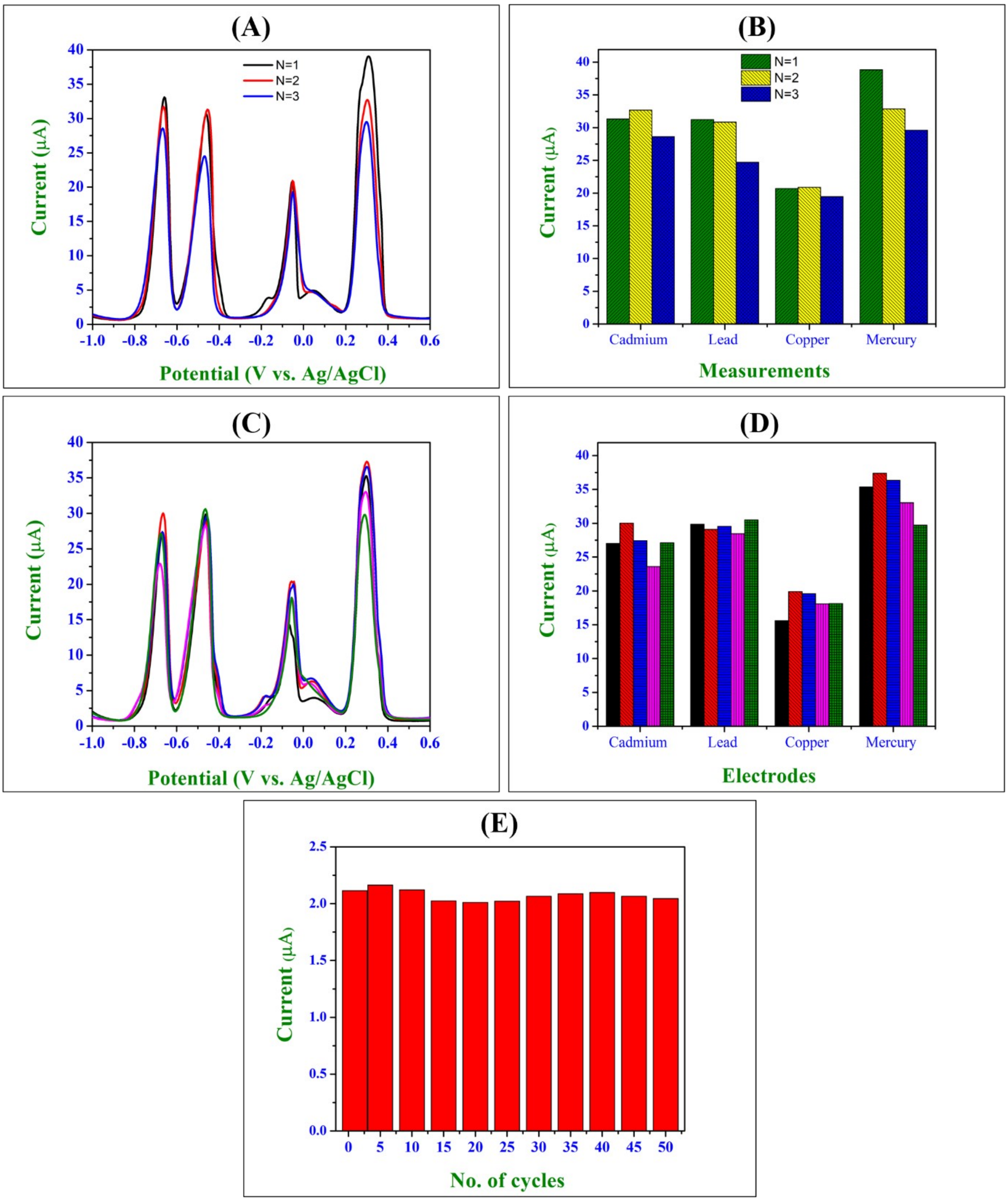

Figure S4. (A) SWASV voltammograms depicting the repeatability measurements for the electrochemical detection of heavy metal ions (Cd^{2+}, Pb^{2+}, Cu^{2+}, and Hg^{2+}) using a $BiVO_4$/GCE. (B) Statistical representation illustrating the correlation between the number of repeated measurements and the corresponding current responses obtained from the repeatability study. (C) SWASV voltammograms demonstrating the reproducibility assessment, where

independently modified $BiVO_4$/GCE electrodes were utilized for the detection of heavy metal ions under identical conditions. (D) Statistical representation depicting the relationship between the number of modified electrodes and the resulting current responses derived from the reproducibility data. (E) The 50 cycles stability of the $BiVO_4$ modified GCE by SWASV.

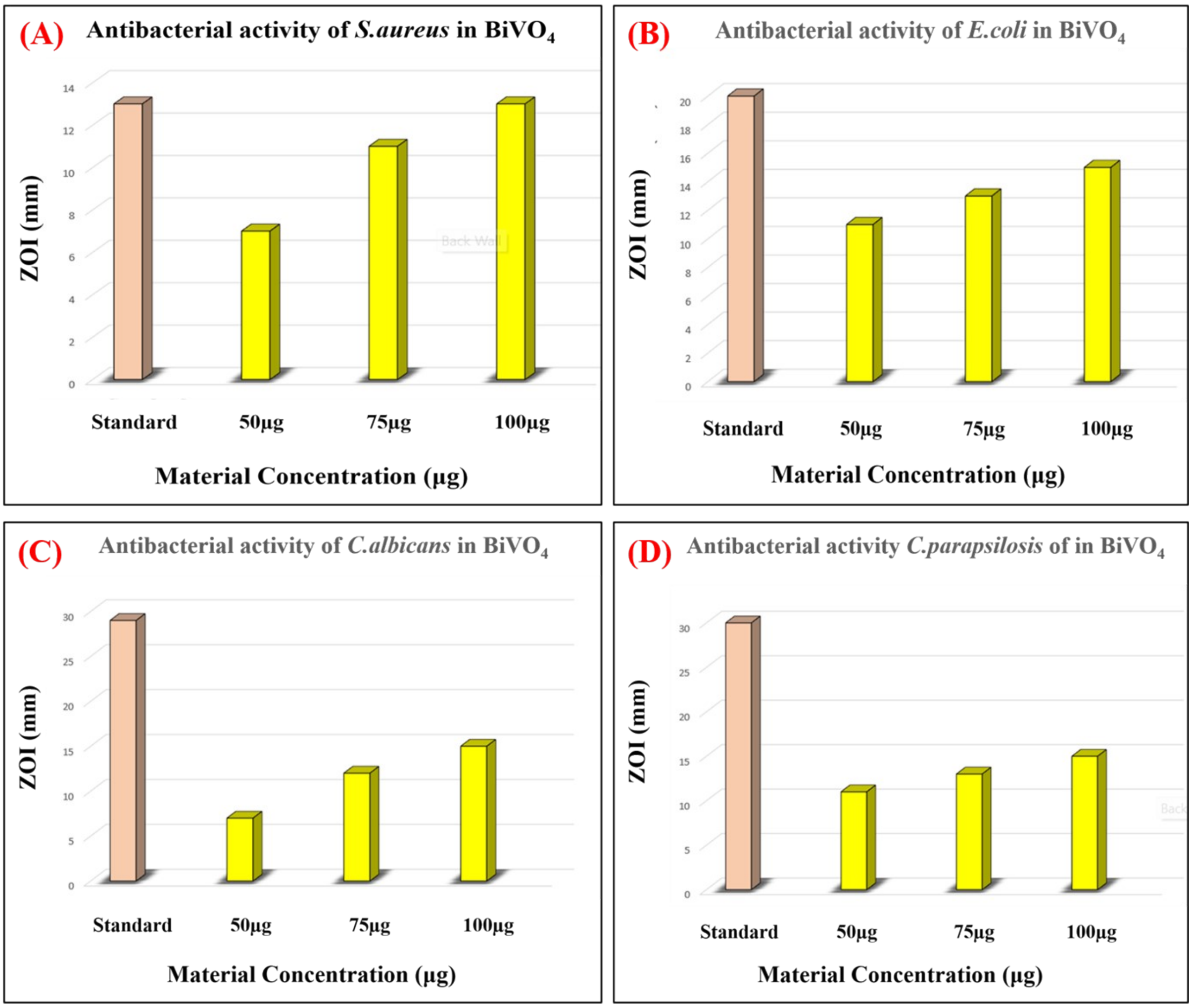

Figure S5. Statistical analysis of the ZOI for various concentrations of $BiVO_4$ nanospheres. The figure provides a detailed comparison and evaluation of the antimicrobial efficacy at different Concentrations 50µl(50µg), 75µl(75µg) and 100µl(100µg) supported by statistical interpretation of the results.

Table S1. Comparative Electrochemical Detection Methods for Heavy Metals Employing Various Sensors Versus Our Proposed Approach.

S.no	Type of electrode	Type of buffer	Material	Technique	Heavy metals	Linear range (μM)	Limit of detection (μM)	Reference
**1**	GCE	PBS	Amino-functionalized mesoporous silica (MCM-41-NH_2 and SBA-15-NH_2)	SWASV	Cd^{2+}	1-100	Between 0.36 - 1.68	[1]
2	GCE	Acetate	Alkaline intercalation of Ti_3C_2 MXene	SWASV	Cd^{2+}, Pb^{2+}, Cu^{2+} and Hg^{2+}	0.1 to 1.5 μM	0.098, 0.041, 0.032 and 0.130	[2]
3	-	PBS	NENE sensors	DPASV	Cu^{2+}	-	0.4	[3]
4	GCE	-	ePdNps@rGO.GCE	LSV	Hg^{2+}	1.0–40	0.33	[4]
5	-	-	Porphyrinic MOF/rGO nanocomposite	DPV	Cu^{2+}	5–150	1.5	[5]

6	-	-	PANI/SWCNTs/SS	DPV	Cu^{2+}	0-2000	1.4	[6]
7	-	-	rGO/MoS_2	DPV	Cd^{2+}, Hg^{2+} & Pb^{2+}	5–160, 5–160 & 10 to 3000 μM	49.83, 36.94, and 733.90	[7]
8	GCE	HEPES	$BiVO_4$/GCE	SWASV	Cd^{2+}, Pb^{2+}, $Cu^{2+}$and Hg^{2+}	0 – 110	2.75 , 2.32, 2.72 and 1.20	This work

Foot notes: DPV- differential pulse voltammetry; DPASV- Differential pulse anodic stripping voltammetry; SPE – screen printed electrode; SWV– Square wave voltammetry; LSV- Linear sweep voltammetry; SWASV- square wave anodic stripping voltammetry; GCE- Glassy carbon electrode; ePdNps/rGO/GCE- reduced graphene oxide and electrodeposited palladium nanoparticles modified GCE; NENE sensors- nanoarray electrode-Nafion-electrode; NHAP nanosized hydroxyapatite; PBS – Phosphate buffer solution; PANI/SWCNTs/SS- Polyaniline/(single walled carbon nanotube)-based composite (PANI/SWCNTs) was electro-deposited on stainless steel (SS); rGO/MoS_2-reduced graphene oxide (RGO)/molybdenum sulfide (MoS_2) composites.

Table S2. The relative standard deviation (RSD) values obtained from the reproducibility analysis for the electrochemical detection of heavy metal ions using $BiVO_4$/GCE.

S.no	Heavy Metals ions	Mean ($\bar{X}$)	Standard Deviation	RSD
1	Cd^{2+}	27.036	2.284366	8.45%
2	Pb^{2+}	29.494	0.768882	2.61%
3	Cu^{2+}	18.256	2.239542	12.37%
4	Hg^{2+}	34.368	3.056104	8.89%

Supporting References

1 A.-M. Sacara, F. Pitzalis, A. Salis, G. L. Turdean and L. M. Muresan, *ACS Omega*, 2019, **4**, 1410–1415.

2 X. Zhu, B. Liu, H. Hou, Z. Huang, K. M. Zeinu, L. Huang, X. Yuan, D. Guo, J. Hu and J. Yang, *Electrochim. Acta*, 2017, **248**, 46–57.

3 L. Zhuo, Y. Huang, M. S. Cheng, H. K. Lee and C.-S. Toh, *Anal. Chem.*, 2010, **82**, 4329–4332.

4 Z. Liu, M. Li, X. Zheng, X. Jia and Y. Guo, *New J Chem*, , DOI:10.1039/d4nj04023f.

5 Y. Huang, Q. Niu, L. Jian, W. Zhao, Y. Li, W. Dong, K. Zhang, W. Liang and C. Yang, *J. Organomet. Chem.*, 2023, **985**, 122597.

6 M. A. Deshmukh, H. K. Patil, G. A. Bodkhe, M. Yasuzawa, P. Koinkar, A. Ramanaviciene, M. D. Shirsat and A. Ramanavicius, *Sens. Actuators B Chem.*, 2018, **260**, 331–338.

7 D. Jiang, K. Sheng, G. Gui, H. Jiang, X. Liu and L. Wang, *Anal. Bioanal. Chem.*, 2021, **413**, 4277–4287.
